# MsQuality: an interoperable open-source package for the calculation of standardized quality metrics of mass spectrometry data

**DOI:** 10.1093/bioinformatics/btad618

**Published:** 2023-10-09

**Authors:** Thomas Naake, Johannes Rainer, Wolfgang Huber

**Affiliations:** Genome Biology Unit, European Molecular Biology Laboratory, Heidelberg 69117, Germany; Institute for Biomedicine (Affiliated to the University of Lübeck), Eurac Research, Bolzano 39100, Italy; Genome Biology Unit, European Molecular Biology Laboratory, Heidelberg 69117, Germany

## Abstract

**Motivation:**

Multiple factors can impact accuracy and reproducibility of mass spectrometry data. There is a need to integrate quality assessment and control into data analytic workflows.

**Results:**

The MsQuality package calculates 43 low-level quality metrics based on the controlled mzQC vocabulary defined by the HUPO-PSI on a single mass spectrometry-based measurement of a sample. It helps to identify low-quality measurements and track data quality. Its use of community-standard quality metrics facilitates comparability of quality assessment and control (QA/QC) criteria across datasets.

**Availability and implementation:**

The R package MsQuality is available through Bioconductor at https://bioconductor.org/packages/MsQuality.

## 1 Introduction 

Mass spectrometry (MS) is a versatile analytical technique that has been adopted in a variety of disciplines, including proteomics, metabolomics, and lipidomics, enabling the identification and quantification of a wide range of molecules. Obtaining high-quality data from mass spectrometry experiments can be a challenging task, as numerous factors can impact the accuracy and reproducibility of the obtained data. To ensure that MS data are fit for purpose, quality assessment and quality control (QA/QC) need to be performed close to data production from raw data (Köcher *et al.* 2011, Bereman 2015). Use of standardized quality metrics described by a controlled vocabulary helps in making QA/QC more comparable across datasets and data producers and increases transparency and trustworthiness of such measures as viewed by data users (Mayer *et al.* 2013, 2014).

**Figure 1.**
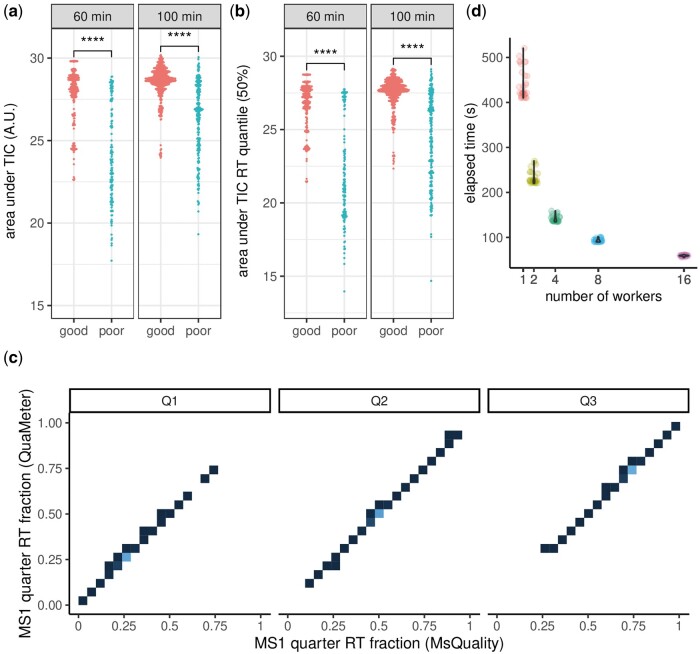
Examples of MsQuality functionality. Metrics are based on MS1 spectra; one data point is obtained per MS1 spectrum. (a) Area under TIC: the area under the total ion chromatogram. (b) Quantiles of area under the total ion chromatogram of the retention time (TIC RT), here, the 50% quantile. For (a) and (b), the data points are displayed as log-values in a beeswarm plot and stratified for high-quality and low-quality measurements as classified in Amidan *et al.* (2014). (c) Comparison of quality metrics calculated by MsQuality and QuaMeter: MS1 quarter RT fraction. The data points are displayed as 2D densities. Brighter areas correspond to high 2D density areas. (d) Wall-clock execution time for the calculation of quality metrics of the dataset of Amidan *et al.* (2014) when parallel computing is used (1, 2, 4, 8, and 16 workers). A.U. arbitrary units.

Here, we introduce the MsQuality R package, which provides functionality to calculate, assess, and track quality metrics for mass spectrometry-derived spectral data of a single mass-spectrometry-based measurement of a sample. The package provides 43 of the mzQC quality metrics defined by the Human Proteome Organization-Proteomics Standards Initiative (HUPO-PSI, hupo-psi.github.io/mzQC). Its use of community standards for data representation in mass spectrometry defined by HUPO-PSI facilitates comparison, consistent storage, reporting and exchange of quality metrics and quality control criteria. The metrics are calculated on low-level MS data such as retention times, m/z, and associated intensity values. The package automates tracking and quantification of data quality on a per-measurement basis and helps to integrate these computations in routine workflows, thereby, MsQuality facilitates the identification of measurements with a high occurrence of missing values, ahead-of-time termination of chromatographic runs, low instrument sensitivity, variations in calibration, and batch and confounding effects within datasets (Fig. 1a and b).

Following the definitions by Bittremieux *et al.* (2017), MsQuality focuses on the calculation of inter-experiment metrics, which is a summarization of an intra-experiment metric. Examples for intra-experiment metrics are the chromatogram of the total ion current (TIC) over the retention time. Inter-experiment metrics, on the other hand, facilitate the comparison of multiple MS runs or experiments, e.g. via longitudinal analysis of quality metrics, such as the fractions of the total retention time required to accumulate a given percentile of the TIC.

## 2 Usage scenario and implementation

The versatility of MsQuality in calculating metrics extends to a wide range of applications, from small-scale studies to long-term acquisition of mass spectrometry data, e.g. a core facility running an instrument for months and years. We demonstrate the utility of MsQuality in two case studies: a metabolomics dataset of 180 cancer cell lines obtained by flow injection analysis (Cherkaoui *et al.* 2022) and a proteomics liquid chromatography (LC)-MS dataset of the same control sample (Amidan *et al.* 2014) as instance of a long-term quality control usage scenario.

The values computed by MsQuality agree with those of QuaMeter (Ma *et al.* 2012) (Fig. 1c): after removing zero-length and zero-intensity entries, as is done by QuaMeter, 75% of the 20 compared metrics showed Pearson correlation coefficients over 0.98 and Spearman correlation coefficients over 0.99 (see the Supplementary Data for further details).

Previously developed QC software, such as PTXQC (Bielow *et al.* 2016), pmultiqc (Perez-Riverol *et al.* 2023), QCloud2 (Olivella *et al.* 2021), and QuaMeter, focused their calculation of QC metrics on proteomics data. MsQuality, on the other hand, is agnostic toward the underlying technology (e.g. proteomics, metabolomics, lipidomics). It is implemented as an GPL-3-licensed open-source R package, and integrates seamlessly into the RforMassSpectrometry/Bioconductor infrastructure for MS data analysis. The package builds upon the established Spectra and MsExperiment packages (Rainer *et al.* 2022) to provide and represent the MS data. Thus, MsQuality supports a large variety of data input formats (ranging from mzML, mzXML, CDF, MGF, MSP to some raw vendor file formats, such as Bruker TimsTOF and Thermo raw files) as well as analyses of very large experiments through the use of data representations with low memory footprint. As MsQuality is written in the software environment R for statistical computing, it facilitates automatized, scalable, easy-to-archive, and shareable scripts for complete data analysis workflows, including pre-processing and statistical analysis steps. Native parallelization enables a fast and scalable calculation of quality metrics (Fig. 1d, see the Supplementary Data for further details).

Besides the human-readable output of quality metrics as a data frame, MsQuality enables the users to export the metrics via the mzQC-defined reporting and exchange file format via the rmzqc package (Bielow 2023). Finally, MsQuality requires little programmatic interaction and is designed to be user-friendly: (i) after the instantiation of Spectra or MsExperiment object, a single function call is needed to calculate the quality metrics; (ii) the metrics can be interactively explored via a shiny application.

## 3 Conclusion

The MsQuality R package provides functionality to calculate, assess, and track quality metrics for mass spectrometry-derived spectral data. It offers easy-to-use means of evaluating data quality, enabling researchers the identification of low-quality measurements. By using standardized quality metrics via the controlled vocabulary of HUPO-PSI, MsQuality helps to make QA/QC more comparable across datasets and data producers. The implementation of MsQuality’s metric calculation is designed to be user-friendly and streamlined and requires little programmatic interaction, facilitating reproducible calculation and evaluation of data quality metrics. MsQuality contributes to the expanding list of tools that use the Spectra/MsExperiment framework (Rainer *et al.* 2022) to address various stages in the analysis pipeline of mass spectrometry data. By building upon this extensive ecosystem for mass spectrometry data, MsQuality enables researchers to create seamless analysis workflows for rapid, efficient, and standardized evaluation of MS data quality, ultimately leading to more robust scientific discoveries in mass spectrometry workflows.

## Supplementary Material

btad618_Supplementary_Data

## Data Availability

The data underlying this article are available in the MassIVE database at https://massive.ucsd.edu via accession number MSV000087155 and in the PRIDE database at https://ebi.ac.uk/pride/ via the accession numbers PXD000320-PXD000324.
