# Supplementary material for: MsQuality: an interoperable open-source package for the calculation of standardized quality metrics of mass spectrometry data
Source: Bioinformatics. 2023 Oct 9;39(10):btad618. doi: 10.1093/bioinformatics/btad618 (PMC10580266; doi:10.1093/bioinformatics/btad618)
Supplement: btad618_Supplementary_Data [file btad618_supplementary_data.pdf]

MsQuality – an interoperable open-source package for the calculation of standardized quality metrics of mass spectrometry data

Supplementary Data

Thomas Naake* Johannes Rainer† Wolfgang Huber‡

Contents

1 Preparation of the environment **3**

2 List of available metrics **4**

3 Quick start to the package **17**

4 Cherkaoui et al. [2022]: A functional analysis of 180 cancer cell lines reveals conserved intrinsic metabolic programs **18**
4.1 Instantiation of the Spectra object . . . 19
4.2 Calculate the metrics via `MsQuality` . . . 19
4.3 Visualization . . . 19
4.4 Performance under parallelization . . . 21

5 Amidan et al. [2014]: Signatures for mass spectrometry data quality **23**
5.1 Instantiation of the Spectra object . . . 23
5.2 Calculate the metrics via `MsQuality` . . . 23
5.3 Visualization . . . 24
5.3.1 Comparison to QuaMeter metrics . . . 31
5.4 Performance under parallelization . . . 41

6 Session info **43**

*Genome Biology Unit, European Molecular Biology Laboratory, Meyerhofstrasse 1, 69117 Heidelberg, Germany

†Institute for Biomedicine (Affiliated Institute of the University of Lübeck), Eurac Research, Viale Druso 1, 39100 Bolzano, Italy

‡Genome Biology Unit, European Molecular Biology Laboratory, Meyerhofstrasse 1, 69117 Heidelberg, Germany

This is the Supplementary Information for the publication "MsQuality - an interoperable open-source package for the calculation of standardized quality metrics of mass spectrometry data". It demonstrates functionality of the `MsQuality` package on two example analysis workflows using the data sets of Cherkaoui et al. [2022] and Amidan et al. [2014].

The `MsQuality` package calculates low-level quality metrics that only require minimal information about the mass spectrometry data: retention time, m/z values, and associated intensities. The list of quality metrics provided by the mzQC framework (hupo-psi.github.io/mzQC) is extensive, also including metrics that depend on higher level information which might not be readily accessible from .raw or .mzML files, such as pump pressure mean, or that rely on alignment results, like retention time mean shift, signal-to-noise ratio, precursor errors (ppm). Such metrics are currently not implemented in `MsQuality`.

The `MsQuality` package relies on the `Spectra` package for data import and representation. Quality metrics are calculated from the information in a `Spectra` object. The `dataOrigin` variable is used to distinguish between the MS data from different measurements/files. Section 1 loads these and other packages into the environment of the `R` session in order to run all analyses.

In subsequent sections of this document the quality of two data sets will be analyzed:

- Section 4: the Cherkaoui et al. [2022] data set is a mass spectrometry (MS) metabolomics data set of 180 cancer cell lines obtained via flow injection analysis (TOF, negative ionization mode). The data set comprises a total of 1397 measurements.
- In Section 5: the Amidan et al. [2014] data set consists of 3431 LC-MS proteomics measurements of a single QC sample (whole cell lysate of *Shewanella oneidensis*). The QC sample was measured on Exactive, LTQ IonTrap, LTQ Orbitrap, and Velos Orbitrap instruments.

We note that these quality metrics are indicative, but by themselves might not be sufficient for data quality control decision-making, such as removing low-quality measurements, which might require additional consideration of more advanced analytics, such as those provided by `MatrixQCvis` [Naake and Huber, 2022]. As stated previously [Bittremieux et al., 2017], the utility of QC metrics depend on the type of the sample, e.g., on whether a single peptide or a complex lysate of proteins is analyzed [Bereman, 2015, Köcher et al. [2011],Paulovich et al. [2010]].

In this document, we will - create `Spectra` objects from the raw data of the two datasets, - calculate the quality metrics on these data sets, - visualize some of the metrics, and - assess performance and scalability of the implemented algorithms using the `microbenchmark` package.

Due to journal's publication format, this document presents static plots. Note that the `MsQuality` package also includes an interactive shiny application to interactively navigate quality metrics, with plots based on the plotly framework. For reproducibility, we provide the source .Rmd file in the accompanying GitHub repository.

A list of the attached packages can be found in Section 6. We will indicate which parts of

this document are reproducible.

1 Preparation of the environment

This analysis uses functions from multiple `R` packages, including `Spectra` for representing mass spectrometry spectral data and `MsQuality` for calculating quality metrics. Other packages are required for data visualization (`ggplot2`, `ggbeeswarm`, `ggpubr`), data wrangling (`dplyr`, `readxl`, `stringr`, `tibble`, `tidyr`), and performance and scalability analysis (`microbenchmark`). Before starting the analysis, ensure to load these packages.

```r
## load packages for visualization
library("ggplot2")
library("ggbeeswarm")
library("ggpubr")

## load packages for data wrangling
library("dplyr")
library("readxl")
library("stringr")
library("tibble")
library("tidyr")

## load packages for performance and scalability analysis
library("microbenchmark")

## load packages for storing spectral data and calculating quality metrics
library("Spectra")
library("MsQuality")
```

2 List of available metrics

The following list gives a brief explanation on the available metrics. Further information may be found at the HUPO-PSI mzQC project page or in the respective help file for the quality metric (accessible by e.g. entering `?chromatographyDuration` to the R console). We also give here explanation on how the metric is calculated in `MsQuality`. Currently, all quality metrics can be calculated for both `Spectra` and `MsExperiment` objects.

- *chromatographyDuration*, **chromatography duration** (MS:4000053), "The retention time duration of the chromatography in seconds." [PSI:MS]; Longer duration may indicate a better chromatographic separation of compounds which depends, however, also on the sampling/scan rate of the MS instrument.

  The metric is calculated as follows:

  (1) the retention time associated to the `Spectra` object is obtained,
  (2) the maximum and the minimum of the retention time is obtained,
  (3) the difference between the maximum and the minimum is calculated and returned.

- *ticQuartersRtFraction*, **TIC quarters RT fraction** (MS:4000054), "The interval when the respective quarter of the TIC accumulates divided by retention time duration." [PSI:MS]; The metric informs about the dynamic range of the acquisition along the chromatographic separation. The metric provides information on the sample (compound) flow along the chromatographic run, potentially revealing poor chromatographic performance, such as the absence of a signal for a significant portion of the run.

  The metric is calculated as follows:

  (1) the `Spectra` object is ordered according to the retention time,
  (2) the cumulative sum of the ion count is calculated (TIC),
  (3) the quantiles are calculated according to the `probs` argument, e.g. when `probs` is set to `c(0, 0.25, 0.5, 0.75, 1)` the 0%, 25%, 50%, 75%, and 100% quantile is calculated,
  (4) the retention time/relative retention time (retention time divided by the total run time taking into account the minimum retention time) is calculated,
  (5) the (relative) duration of the LC run after which the cumulative TIC exceeds (for the first time) the respective quantile of the cumulative TIC is calculated and returned.

- *rtOverMsQuarters*, **MS1 quarter RT fraction** (MS:4000055), "The interval used for acquisition of the first, second, third, and fourth quarter of all MS1 events divided by retention time duration." [PSI:MS], `msLevel = 1L`; The metric informs about the dynamic range of the acquisition along the chromatographic separation. For MS1 scans, the values are expected to be in a similar range across samples of the same type.

  The metric is calculated as follows:

  (1) the retention time duration of the whole `Spectra` object is determined (taking into account all the MS levels),

(2) the `Spectra` object is filtered according to the MS level and subsequently ordered according to the retention time,
(3) the MS events are split into four (approximately) equal parts,
(4) the relative retention time is calculated (using the retention time duration from (1) and taking into account the minimum retention time),
(5) the relative retention time values associated to the MS event parts are returned.

- *rtOverMsQuarters*, **MS2 quarter RT fraction** (MS:4000056), "The interval used for acquisition of the first, second, third, and fourth quarter of all MS2 events divided by retention time duration." [PSI:MS], `msLevel = 2L`; The metric informs about the dynamic range of the acquisition along the chromatographic separation. For MS2 scans, the comparability of the values depends on the acquisition mode and settings to select ions for fragmentation.

  The metric is calculated as follows:

  (1) the retention time duration of the whole `Spectra` object is determined (taking into account all the MS levels),
  (2) the `Spectra` object is filtered according to the MS level and subsequently ordered according to the retention time,
  (3) the MS events are split into four (approximately) equal parts,
  (4) the relative retention time is calculated (using the retention time duration from (1) and taking into account the minimum retention time),
  (5) the relative retention time values associated to the MS event parts are returned.

- *ticQuartileToQuartileLogRatio*, **MS1 TIC-change quartile ratios** (MS:4000057), ""The log ratios of successive TIC-change quartiles. The TIC changes are the list of MS1 total ion current (TIC) value changes from one to the next scan, produced when each MS1 TIC is subtracted from the preceding MS1 TIC. The metric's value triplet represents the log ratio of the TIC-change Q2 to Q1, Q3 to Q2, TIC-change-max to Q3" [PSI:MS], `mode = "TIC_change"`, `relativeTo = "previous"`, `msLevel = 1L`; The metric informs about the dynamic range of the acquisition along the chromatographic separation.This metric evaluates the stability (similarity) of MS1 TIC values from scan to scan along the LC run. High log ratios representing very large intensity differences between pairs of scans might be due to electrospray instability or presence of a chemical contaminant.

  The metric is calculated as follows:

  (1) the TIC (`ionCount`) of the `Spectra` object is calculated per scan event (with spectra ordered by retention time),
  (2) the differences between TIC values are calculated between subsequent scan events,
  (3) the ratios between the 25%, 50%, 75%, and 100% quantile to the 25% quantile of the values of (2) are calculated,
  (4) the `log` values of the ratios are returned.

- *ticQuartileToQuartileLogRatio*, **MS1 TIC quartile ratios** (MS:4000058), "The log ratios of successive TIC quartiles. The metric's value triplet represents the log ratios

of TIC-Q2 to TIC-Q1, TIC-Q3 to TIC-Q2, TIC-max to TIC-Q3.” [PSI:MS], `mode = "TIC"`, `relativeTo = "previous"`, `msLevel = 1L`; The metric informs about the dynamic range of the acquisition along the chromatographic separation. The ratios provide information on the distribution of the TIC values for one LC-MS run. Within an experiment, with the same LC setup, values should be comparable between samples.

The metric is calculated as follows:

(1) the TIC (`ionCount`) of the `Spectra` object is calculated per scan event (with spectra ordered by retention time),
(2) the TIC values between subsequent scan events are taken as they are,
(3) the ratios between the 25%, 50%, 75%, and 100% quantile to the 25% quantile of the values of (2) are calculated,
(4) the `log` values of the ratios are returned.

- *numberSpectra*, **number of MS1 spectra** MS:4000059), “The number of MS1 events in the run.” [PSI:MS], `msLevel = 1L`; An unusual low number may indicate incomplete sampling/scan rate of the MS instrument, low sample volume and/or failed injection of a sample.

  The metric is calculated as follows:

  (1) the `Spectra` object is filtered according to the MS level,
  (2) the number of the spectra are obtained (`length` of `Spectra`) and returned.

- *numberSpectra*, **number of MS2 spectra** (MS:4000060), “The number of MS2 events in the run.” [PSI:MS], `msLevel = 2L`; An unusual low number may indicate incomplete sampling/scan rate of the MS instrument, low sample volume and/or failed injection of a sample.

  The metric is calculated as follows:

  (1) the `Spectra` object is filtered according to the MS level,
  (2) the number of the spectra are obtained (`length` of `Spectra`) and returned.

- *mzAcquisitionRange*, **m/z acquisition range** (MS:4000069), “Upper and lower limit of m/z precursor values at which MSn spectra are recorded.” [PSI:MS]; The metric informs about the dynamic range of the acquisition. Based on the used MS instrument configuration, the values should be similar. Variations between measurements may arise when employing acquisition in DDA mode.

  The metric is calculated as follows:

  (1) the `Spectra` object is filtered according to the MS level,
  (2) the m/z values of the peaks within the `Spectra` object are obtained,
  (3) the minimum and maximum m/z values are obtained and returned.

- *rtAcquisitionRange*, **retention time acquisition range** (MS:4000070), “Upper and lower limit of retention time at which spectra are recorded.” [PSI:MS]; An unusual low range may indicate incomplete sampling and/or a premature or failed LC run.

The metric is calculated as follows:

(1) the `Spectra` object is filtered according to the MS level,
(2) the retention time values of the features within the `Spectra` object are obtained,
(3) the minimum and maximum retention time values are obtained and returned.

- *msSignal10xChange*, **MS1 signal jump (10x) count** (MS:4000097), "The number of times where MS1 TIC increased more than 10-fold between adjacent MS1 scans. An unusual high count of signal jumps or falls can indicate ESI stability issues." [PSI:MS], `change = "jump"`, `msLevel = 1L`; An unusual high count of signal jumps or falls may indicate ESI stability issues.

  The metric is calculated as follows:

  (1) the `Spectra` object is filtered according to the MS level,
  (2) the intensity of the precursor ions within the `Spectra` object are obtained,
  (3) the intensity values of the features are obtained via the ion count,
  (4) the signal jumps/declines of the intensity values with the two subsequent intensity values is calculated,
  (5) the signal jumps by a factor of ten or more are counted and returned.

- *msSignal10xChange*, **MS1 signal fall (10x) count** (MS:4000098), "The number of times where MS1 TIC decreased more than 10-fold between adjacent MS1 scans. An unusual high count of signal jumps or falls can indicate ESI stability issues." [PSI:MS], `change = "fall"`, `msLevel = 1L`; An unusual high count of signal jumps or falls may indicate ESI stability issues.

  The metric is calculated as follows:

  (1) the `Spectra` object is filtered according to the MS level,
  (2) the intensity of the precursor ions within the `Spectra` object are obtained,
  (3) the intensity values of the features are obtained via the ion count,
  (4) the signal jumps/declines of the intensity values with the two subsequent intensity values is calculated,
  (5) the signal declines by a factor of ten or more are counted and returned.

- *numberEmptyScans*, **number of empty MS1 scans** (MS:4000099), "Number of MS1 scans where the scans' peaks intensity sums to 0 (i.e. no peaks or only 0-intensity peaks)." [PSI:MS], `msLevel = 1L`; An unusual high number may indicate incomplete sampling/scan rate of the MS instrument, low sample volume and/or failed injection of a sample.

  The metric is calculated as follows:

  (1) the `Spectra` object is filtered according to the MS level,
  (2) the intensities per entry are obtained,
  (3) the number of intensity entries that are `NULL`, `NA`, or that have a sum of `0` are obtained and returned.

- *numberEmptyScans*, **number of empty MS2 scans** (MS:4000100), "Number of MS2

scans where the scans' peaks intensity sums to 0 (i.e. no peaks or only 0-intensity peaks)." [PSI:MS], `msLevel = 2L`; An unusual high number may indicate incomplete sampling/scan rate of the MS instrument, low sample volume and/or failed injection of a sample.

The metric is calculated as follows:

(1) the `Spectra` object is filtered according to the MS level,
(2) the intensities per entry are obtained,
(3) the number of intensity entries that are `NULL`, `NA`, or that have a sum of `0` are obtained and returned.

- *numberEmptyScans*, **number of empty MS3 scans** (MS:4000101), "Number of MS3 scans where the scans' peaks intensity sums to 0 (i.e. no peaks or only 0-intensity peaks)." [PSI:MS], `msLevel = 3L`; An unusual high number may indicate incomplete sampling/scan rate of the MS instrument, low sample volume and/or failed injection of a sample.

  The metric is calculated as follows:

  (1) the `Spectra` object is filtered according to the MS level,
  (2) the intensities per entry are obtained,
  (3) the number of intensity entries that are `NULL`, `NA`, or that have a sum of `0` are obtained and returned.

- *precursorIntensityQuartiles*, **MS2 precursor intensity distribution Q1, Q2, Q3** (MS:4000116), "From the distribution of MS2 precursor intensities, the quartiles Q1, Q2, Q3." [PSI:MS], `identificationLevel = "all"`; The intensity distribution of the precursors informs about the dynamic range of the acquisition.

  The metric is calculated as follows:

  (1) the `Spectra` object is filtered according to the MS level,
  (2) the intensity of the precursor ions within the `Spectra` object are obtained,
  (3) the 25%, 50%, and 75% quantile of the precursor intensity values are obtained (`NA` values are removed) and returned.

- *precursorIntensityMean*, **MS2 precursor intensity distribution mean** (MS:4000117), "From the distribution of MS2 precursor intensities, the mean." [PSI:MS], `identificationLevel = "all"`; The intensity distribution of the precursors informs about the dynamic range of the acquisition.

  The metric is calculated as follows:

  (1) the `Spectra` object is filtered according to the MS level,
  (2) the intensity of the precursor ions within the `Spectra` object are obtained,
  (3) the mean of the precursor intensity values is obtained (`NA` values are removed) and returned.

- *precursorIntensitySd*, **MS2 precursor intensity distribution sigma** (MS:4000118), "From the distribution of MS2 precursor intensities, the sigma value." [PSI:MS],

`identificationLevel = "all"`; The intensity distribution of the precursors informs about the dynamic range of the acquisition.

The metric is calculated as follows:

(1) the `Spectra` object is filtered according to the MS level,
(2) the intensity of the precursor ions within the `Spectra` object are obtained,
(3) the standard deviation of precursor intensity values is obtained (`NA` values are removed) and returned.

- *medianPrecursorMz*, **MS2 precursor median m/z of identified quantification data points** (MS:4000152), "Median m/z value for MS2 precursors of all quantification data points after user-defined acceptance criteria are applied. These data points may be for example XIC profiles, isotopic pattern areas, or reporter ions (see MS:1001805). The used type should be noted in the metadata or analysis methods section of the recording file for the respective run. In case of multiple acceptance criteria (FDR) available in proteomics, PSM-level FDR should be used for better comparability." [PSI:MS], `identificationLevel = "identified"`, `msLevel = 1L`; The m/z distribution informs about the dynamic range of the acquisition.

  The metric is calculated as follows:

  (1) the `Spectra` object is filtered according to the MS level,
  (2) the precursor m/z values are obtained,
  (3) the median value is returned (`NA`s are removed).

- *rtIqr*, **interquartile RT period for identified quantification data points** (MS:4000153), "The interquartile retention time period, in seconds, for all quantification data points after user-defined acceptance criteria are applied over the complete run. These data points may be for example XIC profiles, isotopic pattern areas, or reporter ions (see MS:1001805). The used type should be noted in the metadata or analysis methods section of the recording file for the respective run. In case of multiple acceptance criteria (FDR) available in proteomics, PSM-level FDR should be used for better comparability." [PSI:MS], `identificationLevel = "identified"`; Longer duration may indicate a better chromatographic separation of compounds which depends, however, also on the sampling/scan rate of the MS instrument.

  The metric is calculated as follows:

  (1) the `Spectra` object is filtered according to the MS level,
  (2) the retention time values are obtained,
  (3) the interquartile range is obtained from the values and returned (`NA` values are removed).

- *rtIqrRate*, **rate of the interquartile RT period for identified quantification data points** (MS:4000154), "The rate of identified quantification data points for the interquartile retention time period, in identified quantification data points per second. These data points may be for example XIC profiles, isotopic pattern areas, or reporter ions (see MS:1001805). The used type should be noted in the metadata or

analysis methods section of the recording file for the respective run. In case of multiple acceptance criteria (FDR) available in proteomics, PSM-level FDR should be used for better comparability." [PSI:MS], `identificationLevel = "identified"`; Higher rates may indicate a more efficient sampling and identification.

The metric is calculated as follows:

(1) the `Spectra` object is filtered according to the MS level,
(2) the retention time values are obtained,
(3) the 25% and 75% quantiles are obtained from the retention time values (`NA` values are removed),
(4) the number of eluted features between this 25% and 75% quantile is calculated,
(5) the number of features is divided by the interquartile range of the retention time and returned.

- *areaUnderTic*, **area under TIC** (MS:4000155), "The area under the total ion chromatogram." [PSI:MS]; The metric informs about the dynamic range of the acquisition. Differences between samples of an experiment may indicate differences in the dynamic range and/or in the sample content.

  The metric is calculated as follows:

  (1) the `Spectra` object is filtered according to the MS level,
  (2) the sum of the ion counts are obtained and returned.

- *areaUnderTicRtQuantiles*, **area under TIC RT quantiles** (MS:4000156), "The area under the total ion chromatogram of the retention time quantiles. Number of quantiles are given by the n-tuple." [PSI:MS]; The metric informs about the dynamic range of the acquisition. Differences between samples of an experiment may indicate differences in the dynamic range and/or in the sample content. The metric informs about the dynamic range of the acquisition along the chromatographic separation. Differences between samples of an experiment may indicate differences in chromatographic performance, differences in the dynamic range and/or in the sample content.

  The metric is calculated as follows:

  (1) the `Spectra` object is filtered according to the MS level,
  (2) the `Spectra` object is ordered according to the retention time,
  (3) the 0%, 25%, 50%, 75%, and 100% quantiles of the retention time values are obtained,
  (4) the ion count of the intervals between the 0%/25%, 25%/50%, 50%/75%, and 75%/100% are obtained,

  (5) the ion counts of the intervals are summed (TIC) and the values returned.

- *extentIdentifiedPrecursorIntensity*, **extent of identified MS2 precursor intensity** (MS:4000157), "Ratio of 95th over 5th percentile of MS2 precursor intensity for all quantification data points after user-defined acceptance criteria are applied. The used type of identification should be noted in the metadata or analysis methods section

of the recording file for the respective run. In case of multiple acceptance criteria (FDR) available in proteomics, PSM-level FDR should be used for better comparability." [PSI:MS], `identificationLevel = "identified"`; The metric informs about the dynamic range of the acquisition.

The metric is calculated as follows:

(1) the `Spectra` object is filtered according to the MS level,
(2) the intensities of the precursor ions are obtained,
(3) the 5% and 95% quantile of these intensities are obtained (`NA` values are removed),
(4) the ratio between the 95% and the 5% intensity quantile is calculated and returned.

- *medianTicRtIqr*, **median of TIC values in the RT range in which the middle half of quantification data points are identified** (MS:4000158), "Median of TIC values in the RT range in which half of quantification data points are identified (RT values of Q1 to Q3 of identifications). These data points may be for example XIC profiles, isotopic pattern areas, or reporter ions (see MS:1001805). The used type should be noted in the metadata or analysis methods section of the recording file for the respective run. In case of multiple acceptance criteria (FDR) available in proteomics, PSM-level FDR should be used for better comparability." [PSI:MS], `identificationLevel = "identified"`; The metric informs about the dynamic range of the acquisition along the chromatographic separation.

  The metric is calculated as follows:

  (1) the `Spectra` object is filtered according to the MS level,
  (2) the `Spectra` object is ordered according to the retention time,
  (3) the features between the 1st and 3rd quartile are obtained (half of the features that are present in the `Spectra` object),
  (4) the ion count of the features within the 1st and 3rd quartile is obtained,
  (5) the median value of the ion count is calculated (`NA` values are removed) and the median value is returned.

- *medianTicOfRtRange*, **median of TIC values in the shortest RT range in which half of the quantification data points are identified** (MS:4000159), "Median of TIC values in the shortest RT range in which half of the quantification data points are identified. These data points may be for example XIC profiles, isotopic pattern areas, or reporter ions (see MS:1001805). The used type should be noted in the metadata or analysis methods section of the recording file for the respective run. In case of multiple acceptance criteria (FDR) available in proteomics, PSM-level FDR should be used for better comparability." [PSI:MS], `identificationLevel = "identified"`; The metric informs about the dynamic range of the acquisition along the chromatographic separation.

  The metric is calculated as follows:

  (1) the `Spectra` object is filtered according to the MS level,
  (2) the `Spectra` object is ordered according to the retention time,

(3) the number of features in the `Spectra` object is obtained and the number for half of the features is calculated,
(4) iterate through the features (always by taking the neighbouring half of features) and calculate the retention time range of the set of features,
(5) retrieve the set of features with the minimum retention time range,
(6) calculate from the set of (5) the median TIC (`NA` values are removed) and return it.

- *precursorIntensityRange*, **MS2 precursor intensity range** (MS:4000160), "Minimum and maximum MS2 precursor intensity recorded." [PSI:MS]; The metric informs about the dynamic range of the acquisition.

  The metric is calculated as follows:

  (1) the `Spectra` object is filtered according to the MS level,
  (2) the intensity of the precursor ions within the `Spectra` object are obtained,
  (3) the minimum and maximum precursor intensity values are obtained and returned.

- *precursorIntensityQuartiles*, **identified MS2 precursor intensity distribution Q1, Q2, Q3** (MS:4000161), "From the distribution of identified MS2 precursor intensities, the quartiles Q1, Q2, Q3. The used type of identification should be noted in the metadata or analysis methods section of the recording file for the respective run. In case of multiple acceptance criteria (FDR) available in proteomics, PSM-level FDR should be used for better comparability." [PSI:MS], `identificationLevel = "identified"`; The metric informs about the dynamic range of the acquisition in relation to identifiability.

  The metric is calculated as follows:

  (1) the `Spectra` object is filtered according to the MS level,
  (2) the intensity of the precursor ions within the `Spectra` object are obtained,
  (3) the 25%, 50%, and 75% quantile of the precursor intensity values are obtained (`NA` values are removed) and returned.

- *precursorIntensityQuartiles*, **unidentified MS2 precursor intensity distribution Q1, Q2, Q3** (MS:4000162), "From the distribution of unidentified MS2 precursor intensities, the quartiles Q1, Q2, Q3. The used type of identification should be noted in the metadata or analysis methods section of the recording file for the respective run. In case of multiple acceptance criteria (FDR) available in proteomics, PSM-level FDR should be used for better comparability." [PSI:MS], `identificationLevel = "unidentified"`; The metric informs about the dynamic range of the acquisition in relation to identifiability.

  The metric is calculated as follows:

  (1) the `Spectra` object is filtered according to the MS level,
  (2) the intensity of the precursor ions within the `Spectra` object are obtained,
  (3) the 25%, 50%, and 75% quantile of the precursor intensity values are obtained (`NA` values are removed) and returned.

- *precursorIntensityMean*, **identified MS2 precursor intensity distribution mean** (MS:4000163), "From the distribution of identified MS2 precursor intensities, the mean. The intensity distribution of the identified precursors informs about the dynamic range of the acquisition in relation to identifiability. The used type of identification should be noted in the metadata or analysis methods section of the recording file for the respective run. In case of multiple acceptance criteria (FDR) available in proteomics, PSM-level FDR should be used for better comparability." [PSI:MS], `identificationLevel = "identified"`; The metric informs about the dynamic range of the acquisition in relation to identifiability.

  The metric is calculated as follows:

  (1) the `Spectra` object is filtered according to the MS level,
  (2) the intensity of the precursor ions within the `Spectra` object are obtained,
  (3) the mean of the precursor intensity values is obtained (`NA` values are removed) and returned.

- *precursorIntensityMean*, **unidentified MS2 precursor intensity distribution mean** (MS:4000164), "From the distribution of unidentified MS2 precursor intensities, the mean. The used type of identification should be noted in the metadata or analysis methods section of the recording file for the respective run. In case of multiple acceptance criteria (FDR) available in proteomics, PSM-level FDR should be used for better comparability." [PSI:MS], `identificationLevel = "unidentified"`; The metric informs about the dynamic range of the acquisition in relation to identifiability.

  The metric is calculated as follows:

  (1) the `Spectra` object is filtered according to the MS level,
  (2) the intensity of the precursor ions within the `Spectra` object are obtained,
  (3) the mean of the precursor intensity values is obtained (`NA` values are removed) and returned.

- *precursorIntensitySd*, **identified MS2 precursor intensity distribution sigma** (MS:4000165), "From the distribution of identified MS2 precursor intensities, the sigma value. The used type of identification should be noted in the metadata or analysis methods section of the recording file for the respective run. In case of multiple acceptance criteria (FDR) available in proteomics, PSM-level FDR should be used for better comparability." [PSI:MS], `identificationLevel = "identified"`; The metric informs about the dynamic range of the acquisition in relation to identifiability.

  The metric is calculated as follows:

  (1) the `Spectra` object is filtered according to the MS level,
  (2) the intensity of the precursor ions within the `Spectra` object are obtained,
  (3) the standard deviation of precursor intensity values is obtained (`NA` values are removed) and returned.

- *precursorIntensitySD*, **unidentified MS2 precursor intensity distribution sigma** (MS:4000166), "From the distribution of unidentified MS2 precursor intensities, the

sigma value. The used type of identification should be noted in the metadata or analysis methods section of the recording file for the respective run. In case of multiple acceptance criteria (FDR) available in proteomics, PSM-level FDR should be used for better comparability." [PSI:MS], `identificationLevel = "unidentified"`; The metric informs about the dynamic range of the acquisition in relation to identifiability.

The metric is calculated as follows:

(1) the `Spectra` object is filtered according to the MS level,
(2) the intensity of the precursor ions within the `Spectra` object are obtained,
(3) the standard deviation of precursor intensity values is obtained (`NA` values are removed) and returned.

- *ratioCharge1over2*, **ratio of 1+ over 2+ of all MS2 known precursor charges** (MS:4000167), "The ratio of 1+ over 2+ MS2 precursor charge count of all spectra." [PSI:MS], `identificationLevel = "all"`; High ratios of 1+/2+ MS2 precursor charge count may indicate inefficient ionization.

  The metric is calculated as follows:

  (1) the `Spectra` object is filtered according to the MS level,
  (2) the precursor charge is obtained,
  (3) the number of precursors with charge 1+ is divided by the number of precursors with charge 2+ and the ratio is returned.

- *ratioCharge1over2*, **ratio of 1+ over 2+ of identified MS2 known precursor charges** (MS:4000168), ""The ratio of 1+ over 2+ MS2 precursor charge count of identified spectra. The used type of identification should be noted in the metadata or analysis methods section of the recording file for the respective run. In case of multiple acceptance criteria (FDR) available in proteomics, PSM-level FDR should be used for better comparability." [PSI:MS], `identificationLevel = "identified"`; High ratios of 1+/2+ MS2 precursor charge count may indicate inefficient ionization in relation to identifiability.

  The metric is calculated as follows:

  (1) the `Spectra` object is filtered according to the MS level,
  (2) the precursor charge is obtained,
  (3) the number of precursors with charge 1+ is divided by the number of precursors with charge 2+ and the ratio is returned.

- *ratioCharge3over2*, **ratio of 3+ over 2+ of all MS2 known precursor charges** (MS:4000169), "The ratio of 3+ over 2+ MS2 precursor charge count of all spectra." [PSI:MS], `identificationLevel = "all"`; Higher ratios of 3+/2+ MS2 precursor charge count may indicate e.g. preference for longer peptides.

  The metric is calculated as follows:

  (1) the `Spectra` object is filtered according to the MS level,
  (2) the precursor charge is obtained,

(3) the number of precursors with charge 3+ is divided by the number of precursors with charge 2+ and the ratio is returned.

- *ratioCharge3over2*, **ratio of 3+ over 2+ of identified MS2 known precursor charges** (MS:4000170), "The ratio of 3+ over 2+ MS2 precursor charge count of identified spectra. The used type of identification should be noted in the metadata or analysis methods section of the recording file for the respective run. In case of multiple acceptance criteria (FDR) available in proteomics, PSM-level FDR should be used for better comparability." [PSI:MS], `identificationLevel = "identified"`; Higher ratios of 3+/2+ MS2 precursor charge count may indicate e.g. preference for longer peptides in relation to identifiability.

  The metric is calculated as follows:

  (1) the `Spectra` object is filtered according to the MS level,
  (2) the precursor charge is obtained,
  (3) the number of precursors with charge 3+ is divided by the number of precursors with charge 2+ and the ratio is returned.

- *ratioCharge4over2*, **ratio of 4+ over 2+ of all MS2 known precursor charges** (MS:4000171), "The ratio of 4+ over 2+ MS2 precursor charge count of all spectra." [PSI:MS], `identificationLevel = "all"`; Higher ratios of 3+/2+ MS2 precursor charge count may indicate e.g. preference for longer peptides.

  The metric is calculated as follows:

  (1) the `Spectra` object is filtered according to the MS level,
  (2) the precursor charge is obtained,
  (3) the number of precursors with charge 4+ is divided by the number of precursors with charge 2+ and the ratio is returned.

- *ratioCharge4over2*, **ratio of 4+ over 2+ of identified MS2 known precursor charges** (MS:4000172), "The ratio of 4+ over 2+ MS2 precursor charge count of identified spectra. The used type of identification should be noted in the metadata or analysis methods section of the recording file for the respective run. In case of multiple acceptance criteria (FDR) available in proteomics, PSM-level FDR should be used for better comparability." [PSI:MS], `identificationLevel = "identified"`; Higher ratios of 3+/2+ MS2 precursor charge count may indicate e.g. preference for longer peptides in relation to identifiability.

  The metric is calculated as follows:

  (1) the `Spectra` object is filtered according to the MS level,
  (2) the precursor charge is obtained,
  (3) the number of precursors with charge 4+ is divided by the number of precursors with charge 2+ and the ratio is returned.

- *meanCharge*, **mean MS2 precursor charge in all spectra** (MS:4000173), "Mean MS2 precursor charge in all spectra" [PSI:MS], `identificationLevel = "all"`; Higher charges may indicate inefficient ionization or e.g. preference for longer peptides.

The metric is calculated as follows:

(1) the `Spectra` object is filtered according to the MS level,
(2) the precursor charge is obtained,
(3) the mean of the precursor charge values is calculated and returned.

- *meanCharge*, **mean MS2 precursor charge in identified spectra** (MS:4000174), "Mean MS2 precursor charge in identified spectra. The used type of identification should be noted in the metadata or analysis methods section of the recording file for the respective run. In case of multiple acceptance criteria (FDR) available in proteomics, PSM-level FDR should be used for better comparability." [PSI:MS], `identificationLevel = "identified"`; Higher charges may indicate inefficient ionization or e.g. preference for longer peptides in relation to identifiability.

  The metric is calculated as follows:

  (1) the `Spectra` object is filtered according to the MS level,
  (2) the precursor charge is obtained,
  (3) the mean of the precursor charge values is calculated and returned.

- *medianCharge*, **median MS2 precursor charge in all spectra** (MS:4000175), "Median MS2 precursor charge in all spectra" [PSI:MS], `identificationLevel = "all"`; Higher charges may indicate inefficient ionization and/or e.g. preference for longer peptides.

  The metric is calculated as follows:

  (1) the `Spectra` object is filtered according to the MS level,
  (2) the precursor charge is obtained,
  (3) the median of the precursor charge values is calculated and returned.

- *medianCharge*, **median MS2 precursor charge in identified spectra** (MS:4000176), "Median MS2 precursor charge in identified spectra. The used type of identification should be noted in the metadata or analysis methods section of the recording file for the respective run. In case of multiple acceptance criteria (FDR) available in proteomics, PSM-level FDR should be used for better comparability." [PSI:MS], `identificationLevel = "identified"`; Higher charges may indicate inefficient ionization and/or e.g. preference for longer peptides in relation to identifiability.

  The metric is calculated as follows:

  (1) the `Spectra` object is filtered according to the MS level,
  (2) the precursor charge is obtained,
  (3) the median of the precursor charge values is calculated and returned.

An up-to-date list can be found in the vignette of the package via Bioconductor.

3 Quick start to the package

For demonstration purposes, we apply here the `MsQuality` package on the .mzML files shipped by the `msdata` package. One of the files is a LC-MS/MS DIA (SWATH) and one a LC-MS/MS DDA file.

```
fls <- dir(system.file("TripleTOF-SWATH", package = "msdata"),
    full.names = TRUE)
```

In a next step, we create a `Spectra` object from the two .mzML files using the `Spectra` function.

```
sps <- Spectra(fls, backend = MsBackendMzR())
```

The `Spectra` object is given to the `calculateMetricsFromSpectra` function. We are interested in the metrics `"chromatographyDuration"`, `"numberSpectra"`, and `"areaUnderTic"`). The metrics are defined by the `metrics` vector. We further specify that the metrics should be calculated on MS1 spectra (`msLevel = 1`).

```
metrics <- c("chromatographyDuration", "numberSpectra", "areaUnderTic")

## calculate the metrics
metrics_sps <- calculateMetricsFromSpectra(spectra = sps, metrics = metrics,
    msLevel = 1)
```

The output of the function is per default a `data.frame` object that has the metrics as columns and the samples as rows.

```
## for visualization purposes the rownames are truncated to the base names
rownames(metrics_sps) <- basename(rownames(metrics_sps))
metrics_sps
```

```
##                        chromatographyDuration numberSpectra areaUnderTic
## PestMix1_DDA.mzML                     899.762          4627    2627432.5
## PestMix1_SWATH.mzML                   899.712           999     540515.4
## attr(,"chromatographyDuration")
## [1] "MS:4000053"
## attr(,"numberSpectra")
## [1] "MS:4000059"
## attr(,"areaUnderTic")
## [1] "MS:4000155"
## attr(,"msLevel")
## [1] 1
```

4 Cherkaoui et al. [2022]: A functional analysis of 180 cancer cell lines reveals conserved intrinsic metabolic programs

The .mzML files were downloaded from the MassIVE database (accession number MSV000087155, available at https://massive.ucsd.edu/) via ftp://massive.ucsd.edu/MSV000087155.

We use the BiocFileCache package from Bioconductor to download and cache the .mzML files locally. To this end we first determine below the full file names of all .mzML files of this data set.

All parts in the section *Cherkaoui et al. [2022]: A functional analysis of 180 cancer cell lines reveals conserved intrinsic metabolic programs* are reproducible except the parallelization steps in the subsection **Performance under parallelization**. For these steps precalculated objects are loaded to the environment.

```
url <- "ftp://massive.ucsd.edu/v01/MSV000087155/ccms_peak/New_mzMLFinal/"
library(curl)
```

```
## Using libcurl 7.84.0 with Schannel
```

```
file_list <- readLines(curl(url, "r"))
ftp_files <- strsplit(file_list, " +")
ftp_files <- vapply(
    ftp_files, function(z) paste0(tail(z, 2), collapse = " "), character(1))
```

With the file names available we next create a *BiocFileCache* instance adding the files. The *BiocFileCache* will take care of downloading files that are not already available in the local cache thus preventing unneeded downloads.

```
library(BiocFileCache)

## every additional result should be saved in there
cherkaoui <- BiocFileCache("../Cherkaoui2022", ask = FALSE)
path <- bfcrpath(cherkaoui, paste0(url, curl_escape(ftp_files)))
```

These downloaded .mzML files can however not be directly loaded because they are not fully compliant with the open .mzML standard file format (internal references to instrumentation configuration are missing). We thus need to process all files to remove these incompatible lines from each .mzML file. This needs to be done (once) using the below unix shell commands that should be executed in the folder containing the downloaded files.

```
cd ../Cherkaoui2022
sed -i 's/<run defaultInstrumentConfigurationRef=.*/<run/g' *.mzML
sed -i '/^<scanWindowList/,/^<\/scanWindowList/d' *.mzML
```

4.1 Instantiation of the Spectra object

In the subsequent analysis, a `Spectra` object is instantiated. The operations were executed within a (high-performance) computing environment (31 cores, 64 GB RAM pool for all cores).

```
## create the Spectra object
sps <- Spectra(path, backend = MsBackendMzR())
```

4.2 Calculate the metrics via `MsQuality`

`MsQuality` uses the `Spectra` class for storing the spectral data. In this particular case, where the spectral data was obtained via flow injection analysis, metrics that incorporate retention time information are not relevant and the analysis will only focus on the three metrics

- *numberSpectra*, **Number of MS1 spectra** (MS:4000059), "The number of MS1 events in the run." [PSI:MS];
- *areaUnderTic*, **Area under TIC** (MS:4000155), "The area under the total ion chromatogram." [PSI:MS];
- *mzAcquisitionRange*, **m/z acquisition range** (MS:4000069), "Upper and lower limit of m/z precursor values at which MSn spectra are recorded." [PSI:MS].

The metrics are calculated using the function `calculateMetricsFromSpectra`, which takes as input the `Spectra` object, `sps`, and the above-defined metrics. Optional parameters can also be passed to this function for further control of the calculation, such as `msLevel` for cases where multiple mass spectra levels are present in the `Spectra` object. It is unnecessary to specify `msLevel` in the current context since only MS1 level spectra are stored in the `Spectra` object.

```
metrics <- c("numberSpectra", "areaUnderTic", "mzAcquisitionRange")

metrics_sps <- calculateMetricsFromSpectra(spectra = sps,
    metrics = metrics)
```

4.3 Visualization

We next visualize the three quality metrics using the `ggplot2` package. We include also information from the original study Cherkaoui et al. [2022] in particular which of the files were included in the final analysis. The results of the study are available from this resource: https://doi.org/10.3929/ethz-b-000511784 . We first download and cache the *PrimaryAnalysis.zip* archive that contains all results, unzip it to a temporary folder and import the *metabolomics_180CCL.xlsx* file.

```
l <- paste0("https://www.research-collection.ethz.ch/bitstream/handle/",
    "20.500.11850/511784/PrimaryAnalysis.zip?sequence=1&isAllowed=y")
arch <- bfcrpath(cherkaoui, l)
```

```
## adding rname 'https://www.research-collection.ethz.ch/bitstream/handle/20.500.11850/5
```

```r
unzip(zipfile = arch, files = "PrimaryAnalysis/Metabolomics_180CCL.xlsx",
    exdir = tempdir())
measurements <- read_xlsx(
    file.path(tempdir(), "PrimaryAnalysis/Metabolomics_180CCL.xlsx"),
    sheet = "injections")
```

From this excel sheet we retrieve the information whether a measurements was analyzed or excluded and add this information to the `metrics_sps` object with the quality information.

We then create a Figure to compare the differences in quality metrics between the analyzed and excluded measurements (Figure S1).

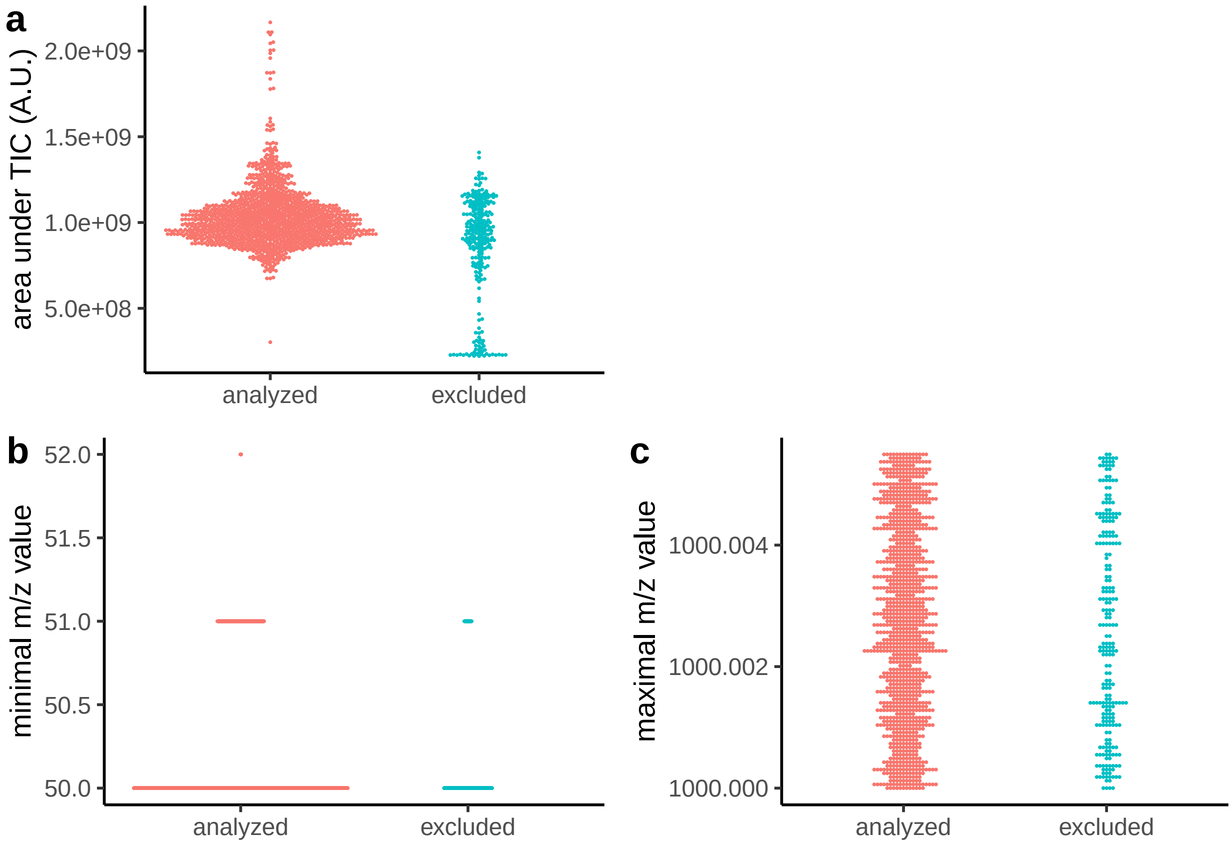

Figure S1: Quality metrics for data set of Cherkaoui et al. [2022] stratified by information if the measurement was analyzed or excluded. (a) Area under the TIC (`areaUnderTic`). (b) Minimum values of the m/z acquisition range (`mzAcquisitionRange.min`). (c) maximum values of the m/z acquisition range `mzAcquisitionRange.max`). A.U.: arbitrary units.

Figure S1 demonstrates that the excluded measurements show a bimodal distribution of the total ion current (TIC). Specifically, some of the excluded measurements have lower total ion current (TIC) values, which was already noted in the original publication and was the reason for their exclusion from subsequent analysis steps. Figure S1 a serves as a visual confirmation of this statement and aids in understanding the data quality of the measurements The metrics

`mzAcquisitionRange.max` and `mzAcquisitionRange.min` on the other hand (Figure S1 (b) and (c)) are not informative for the decision making on excluding/including the measurements in further analysis steps.

4.4 Performance under parallelization

An important aspect, especially when dealing with large amount of data, is scalability and performance when computing the quality metric.

By monitoring parallelization, it is possible to determine the scalability of the computation and ensure that the performance of the analysis remains acceptable as the data size increases.

We measure below the time it takes to evaluate the calculation of quality metrics by parallelizing the tasks on 1, 2, 4, 8, and 16 workers using the `microbenchmark` package. This package allows for precise measurement of the execution time of `R` expressions by repeating the evaluation multiple times and providing detailed summary statistics of the execution times.

```
metrics <- c("numberSpectra", "areaUnderTic", "mzAcquisitionRange")
df_mb <- microbenchmark(calculateMetricsFromSpectra(spectra = sps,
        metrics = metrics, BPPARAM = MulticoreParam(workers = 1)),
    workers_2 = calculateMetricsFromSpectra(spectra = sps,
        metrics = metrics, BPPARAM = MulticoreParam(workers = 2)),
    workers_4 = calculateMetricsFromSpectra(spectra = sps,
        metrics = metrics, BPPARAM = MulticoreParam(workers = 4)),
    workers_8 = calculateMetricsFromSpectra(spectra = sps,
        metrics = metrics, BPPARAM = MulticoreParam(workers = 8)),
    workers_16 = calculateMetricsFromSpectra(spectra = sps,
        metrics = metrics, BPPARAM = MulticoreParam(workers = 16)),
    times = 110L, control = list(warmup = 10), check = "equal"
)
```

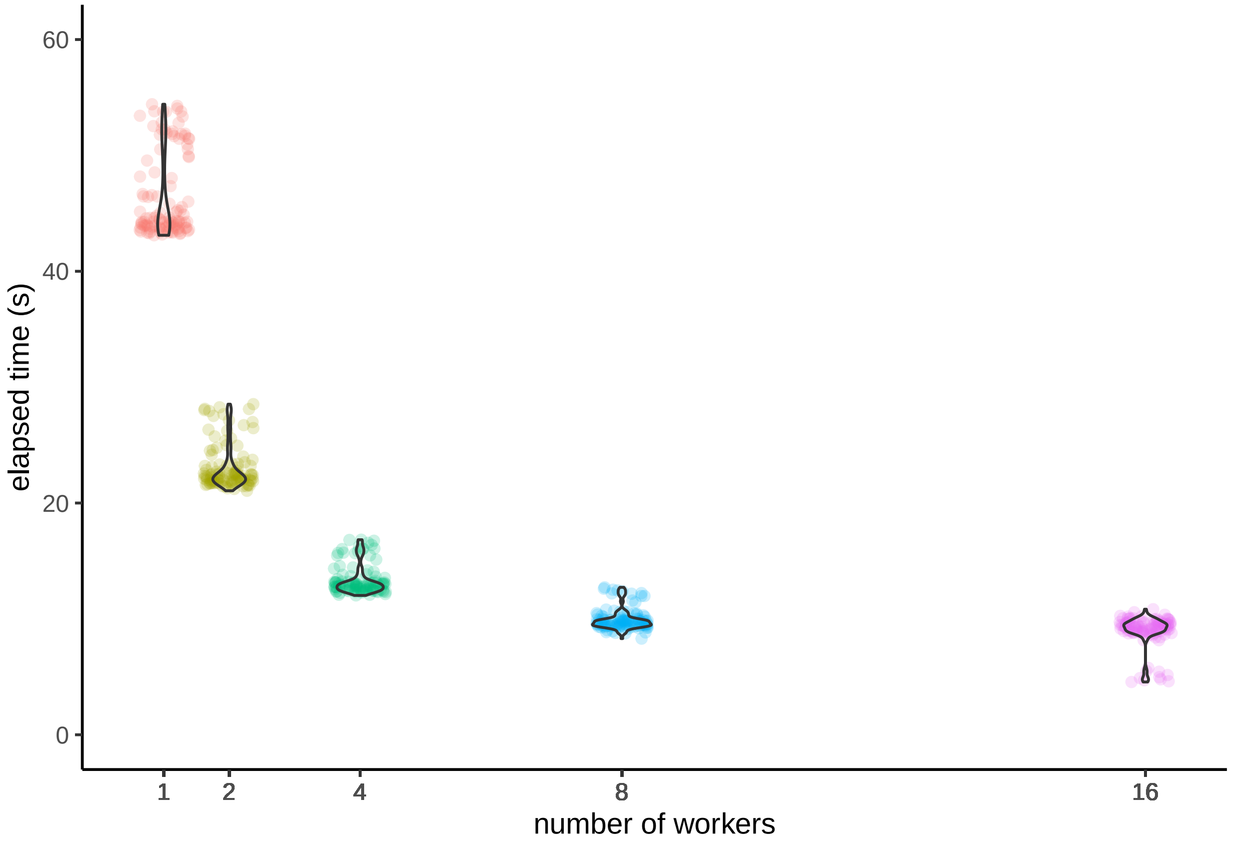

Figure S2: Execution time for the calculation of quality metrics of the data set of Cherkaoui et al. [2022] under parallelization (1, 2, 4, 8, and 16 workers).

By parallelizing the calculation of the quality metrics across multiple workers, it is possible to significantly reduce the execution time, and the `microbenchmark` package was used to accurately measure the performance improvements achieved by parallelization (Figure S2). The parallelization process can help in the management of bigger data sets, and to save valuable time in data analysis.

5 Amidan et al. [2014]: Signatures for mass spectrometry data quality

The RAW files were downloaded from

- ftp://ftp.pride.ebi.ac.uk/pride/data/archive/2013/10/PXD000320 (1_of_5),
- ftp://ftp.pride.ebi.ac.uk/pride/data/archive/2013/10/PXD000321 (2_of_5),
- ftp://ftp.pride.ebi.ac.uk/pride/data/archive/2013/10/PXD000322 (3_of_5),
- ftp://ftp.pride.ebi.ac.uk/pride/data/archive/2013/10/PXD000323 (4_of_5),
- ftp://ftp.pride.ebi.ac.uk/pride/data/archive/2013/10/PXD000324 (5_of_5).

Subsequently, the RAW files were converted into `.mzML` files using MSConvertGUI (64-bit, v3.0.22015-aadd392) with setting `peakPicking` to `vendor msLevel=1-`.

The creation of the Figures in the section *Amidan et al. [2014]: Signatures for mass spectrometry data quality* are reproducible. Due to long computation time or requirement of an environment that enables for parallelization, the creation of the `Spectra` object in the subsection **Instantiation of the Spectra object**, the calculation of the quality metrics in the subsection **Calculate the metrics via `MsQuality`**, and the parallelization steps in the subsection **Performance under parallelization** are precomputed.

5.1 Instantiation of the Spectra object

In the subsequent analysis, a `Spectra` object is instantiated. The operations were executed within a (high-performance) computing environment (3 cores, 128 GB RAM pool for all cores), where the .mzML files were stored in the directory `Amidan2014`.

```
## read the file with protein intensities
path <- "/scratch/naake/Amidan2014"
fls <- dir(path, full.names = TRUE, recursive = TRUE, pattern = "mzML")

## create the Spectra object
sps <- Spectra(fls, backend = MsBackendMzR())
```

5.2 Calculate the metrics via `MsQuality`

`MsQuality` utilizes `Spectra` objects that store the spectral data. Here, retention time information was available from the .mzML files and a higher number of metrics could be calculated.

The metrics are calculated using the function `calculateMetricsFromSpectra`, which takes as input the `Spectra` object, `sps`, and the above-defined metrics. We calculate the metrics separately for the MS1 (`msLevel 1`) and MS2 spectra (`msLevel 2`). In addition, the metrics are calculated on the `Spectra` objects where zero-intensity, `Inf`-intensity, and zero-length entries are removed (`filterEmptySpectra = FALSE`) and where the `Spectra` objects are not filtered (`filterEmptySpectra = TRUE`).

```
metrics <- c("chromatographyDuration", "ticQuartersRtFraction",
    "rtOverMsQuarters", "ticQuartileToQuartileLogRatio", "numberSpectra",
    "numberEmptyScans", "medianPrecursorMz", "rtIqr", "rtIqrRate",
    "areaUnderTic", "areaUnderTicRtQuantiles", "medianTicRtIqr",
    "medianTicOfRtRange", "mzAcquisitionRange", "rtAcquisitionRange",
    "precursorIntensityRange", "precursorIntensityQuartiles",
    "precursorIntensityMean", "precursorIntensitySd", "msSignal10xChange",
    "ratioCharge1over2", "ratioCharge3over2", "ratioCharge4over2",
    "meanCharge", "medianCharge")

## remove zero-intensity and zero-length entries
metrics_sps_msLevel1_filtered <- calculateMetricsFromSpectra(spectra = sps,
    metrics = metrics, filterEmptySpectra = TRUE, msLevel = 1L)
metrics_sps_msLevel1_filtered <- calculateMetricsFromSpectra(spectra = sps,
    metrics = metrics, filterEmptySpectra = TRUE, msLevel = 2L)

## take the entries as they are
metrics_sps_msLevel1 <- calculateMetricsFromSpectra(spectra = sps,
    metrics = metrics, filterEmptySpectra = FALSE, msLevel = 1L)
metrics_sps_msLevel1 <- calculateMetricsFromSpectra(spectra = sps,
    metrics = metrics, filterEmptySpectra = FALSE, msLevel = 2L)
```

Overall, this function provides a flexible and efficient way to analyze large amounts of mass spectrometry data and obtain insights on the quality of the data.

5.3 Visualization

In the analysis of the Amidan et al. [2014] study, the quality metrics were visualized using the `ggplot2` package. The XLS files pr401143e_si_002.xls and pr401143e_si_003.xls (provided as Supplemental Material of the original publication) was used to extract information on the measurement quality. This information was added to the `metrics_sps_msLevel1_filtered`, `metrics_sps_msLevel2_filtered`, `metrics_sps_msLevel1` and `metrics_sps_msLevel2` objects.

The Figures S3, S4, S5, S6, S7, and S8 were created as examples to compare the differences between the low- and high-quality measurements for several of the supported quality metrics. `filtered` refers to the metrics where `filterEmptySpectra` was set to `TRUE`, on the other hand, `unfiltered` refers to the metrics where `filterEmptySpectra` was set to `FALSE`.

While the metric `chromatographyDuration` (retention time) is a continuous variable, for visualization purposes we will bin the variable to discrete values and will use the measurements over 60 min and 100 min for visualization.

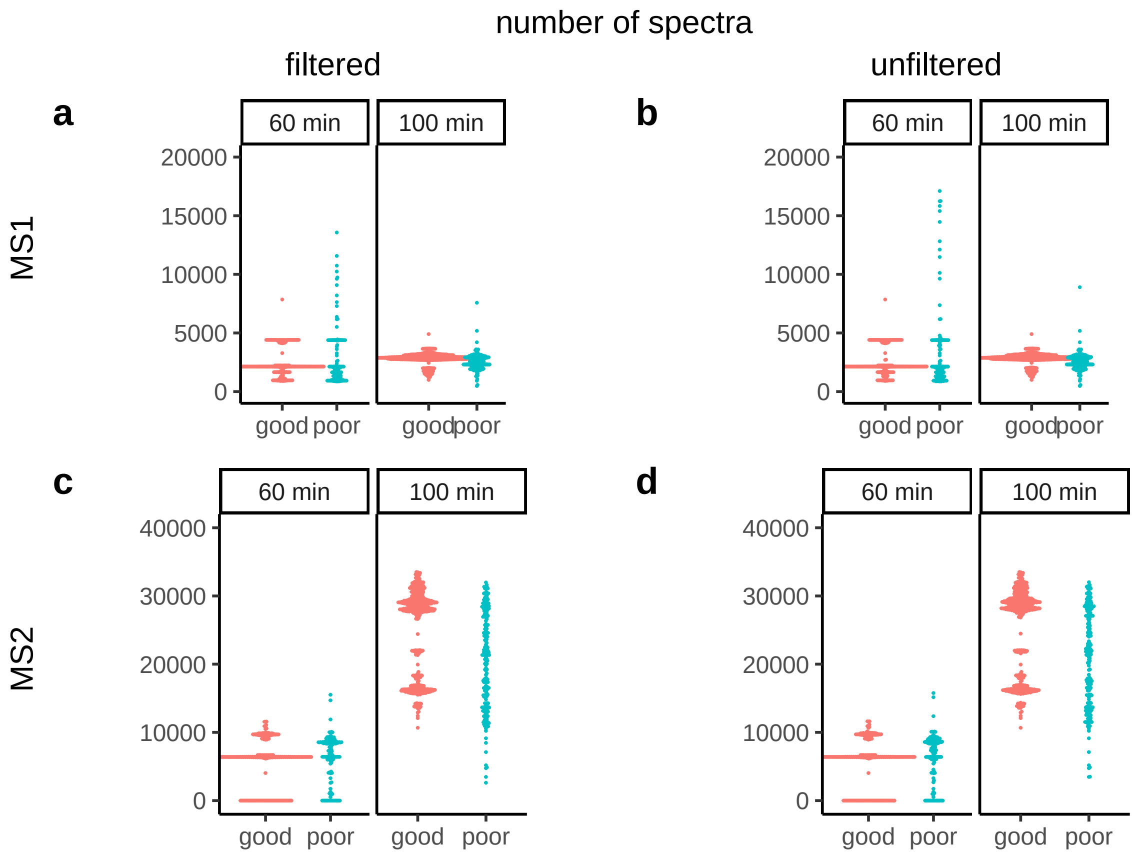

Figure S3: Quality metrics by `MsQuality`: Number of MS1 and MS2 spectra (`numberSpectra`). The `MsQuality` metrics are calculated from filtered and unfiltered MS1 and MS2 spectra. One data point is obtained per MS1 and MS2 measurement run and the data points are displayed as beeswarm plots stratified for high-quality and low-quality measurements as classified in Amidan et al. [2014]. (a) Number of filtered MS1 spectra. (b) Number of unfiltered MS1 spectra. (c) Number of filtered MS2 spectra. (d) Number of unfiltered MS2 spectra.

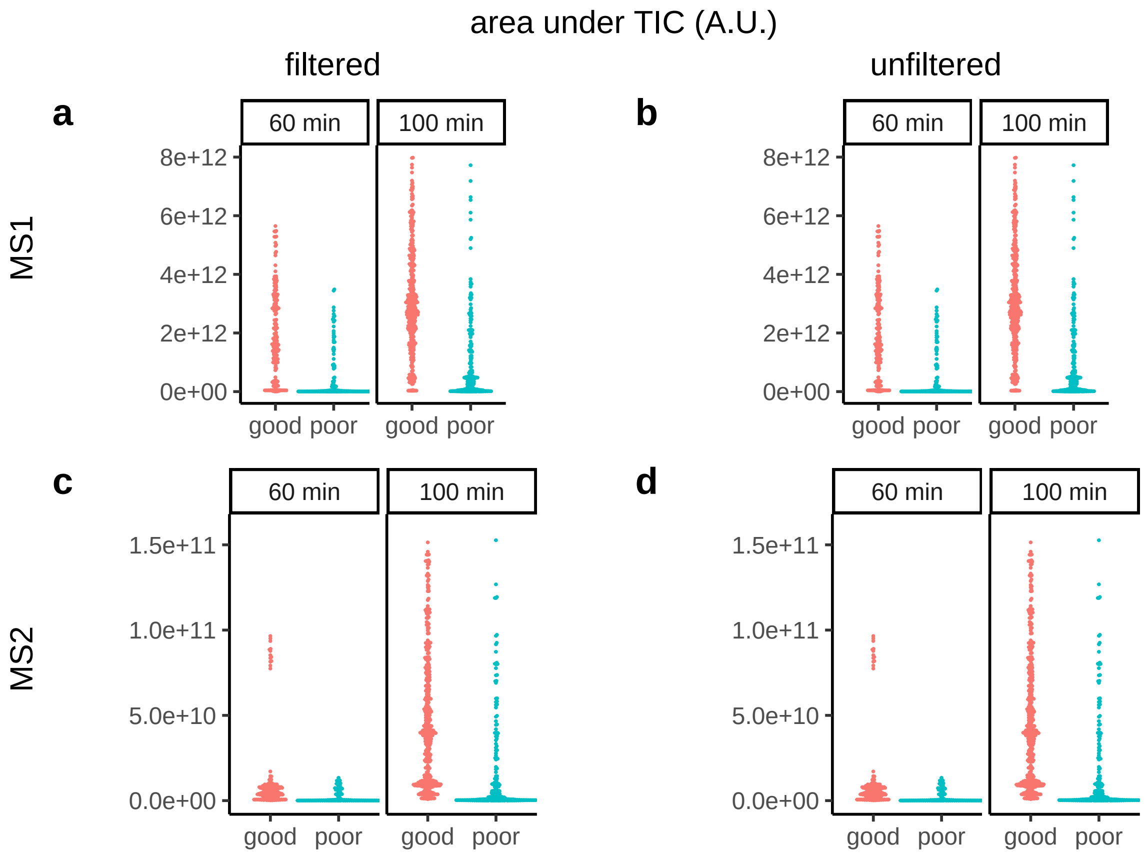

Figure S4: Quality metrics by `MsQuality`: Area under TIC (`areaUnderTic`). The `MsQuality` metrics are calculated from filtered and unfiltered MS1 and MS2 spectra. One data point is obtained per MS1 and MS2 measurement run and the data points are displayed as beeswarm plots stratified for high-quality and low-quality measurements as classified in Amidan et al. [2014]. (a) Area under TIC for filtered MS1 spectra. (b) Area under TIC for unfiltered MS1 spectra. (c) Area under TIC for filtered MS2 spectra. (d) Area under TIC for unfiltered MS2 spectra. A.U.: arbitrary units.

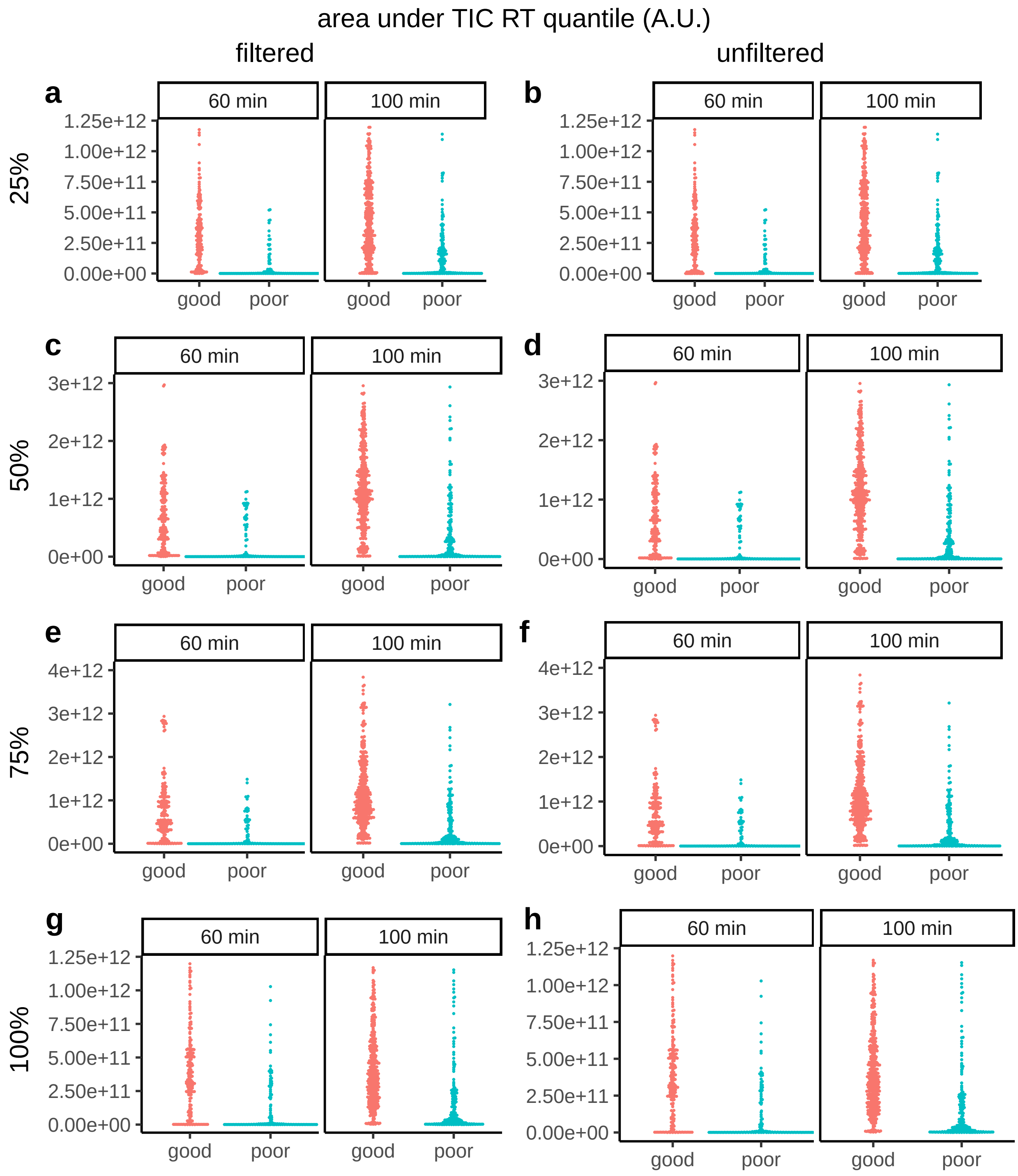

Figure S5: Quality metrics by `MsQuality`: Area under TIC RT quantiles (`areaUnderTicRtQuantiles`). The `MsQuality` metrics are calculated from filtered and unfiltered MS1 spectra. One data point is obtained per MS1 measurement run and the data points are displayed as beeswarm plots stratified for high-quality and low-quality measurements as classified in Amidan et al. [2014]. (a) 25% quantile for filtered MS1 spectra. (b) 25% quantile for unfiltered MS1 spectra. (c) 50% quantile for filtered MS1 spectra. (d) 50% quantile for unfiltered MS1 spectra. (e): 75% quantile for filtered MS1 spectra. (f) 75% quantile for unfiltered MS1 spectra. (g) 100% quantile for filtered MS1 spectra. (h) 100% quantile for unfiltered MS1 spectra.

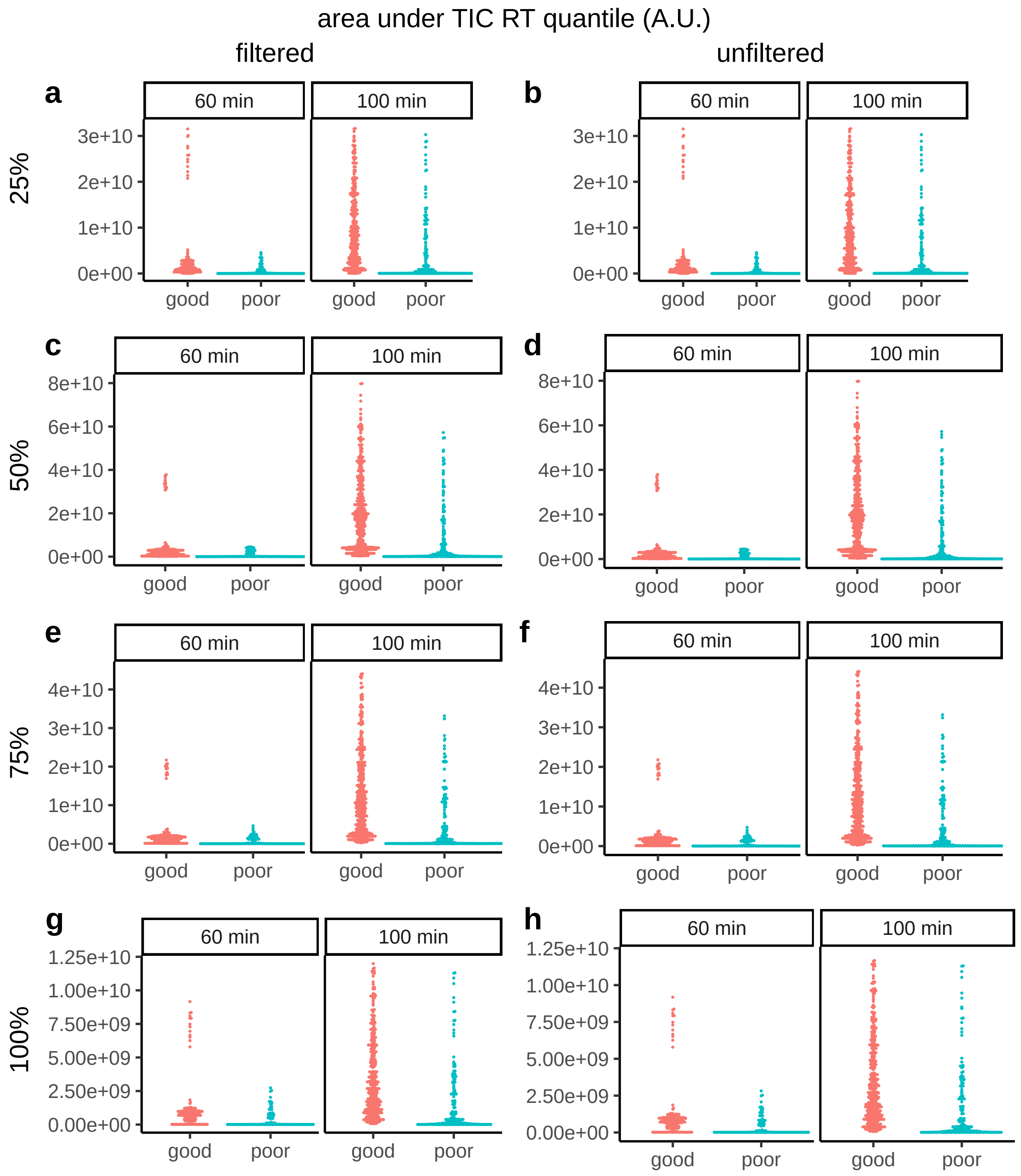

Figure S6: Quality metrics by `MsQuality`: Area under TIC RT quantiles (`areaUnderTicRtQuantiles`). The `MsQuality` metrics are calculated from filtered and unfiltered MS2 spectra. One data point is obtained per MS2 measurement run and the data points are displayed as beeswarm plots stratified for high-quality and low-quality measurements as classified in Amidan et al. [2014]. (a) 25% quantile for filtered MS2 spectra. (b) 25% quantile for unfiltered MS2 spectra. (c) 50% quantile for filtered MS2 spectra. (d) 50% quantile for unfiltered MS2 spectra. (e): 75% quantile for filtered MS2 spectra. (f) 75% quantile for unfiltered MS2 spectra. (g) 100% quantile for filtered MS2 spectra. (h) 100% quantile for unfiltered MS2 spectra.

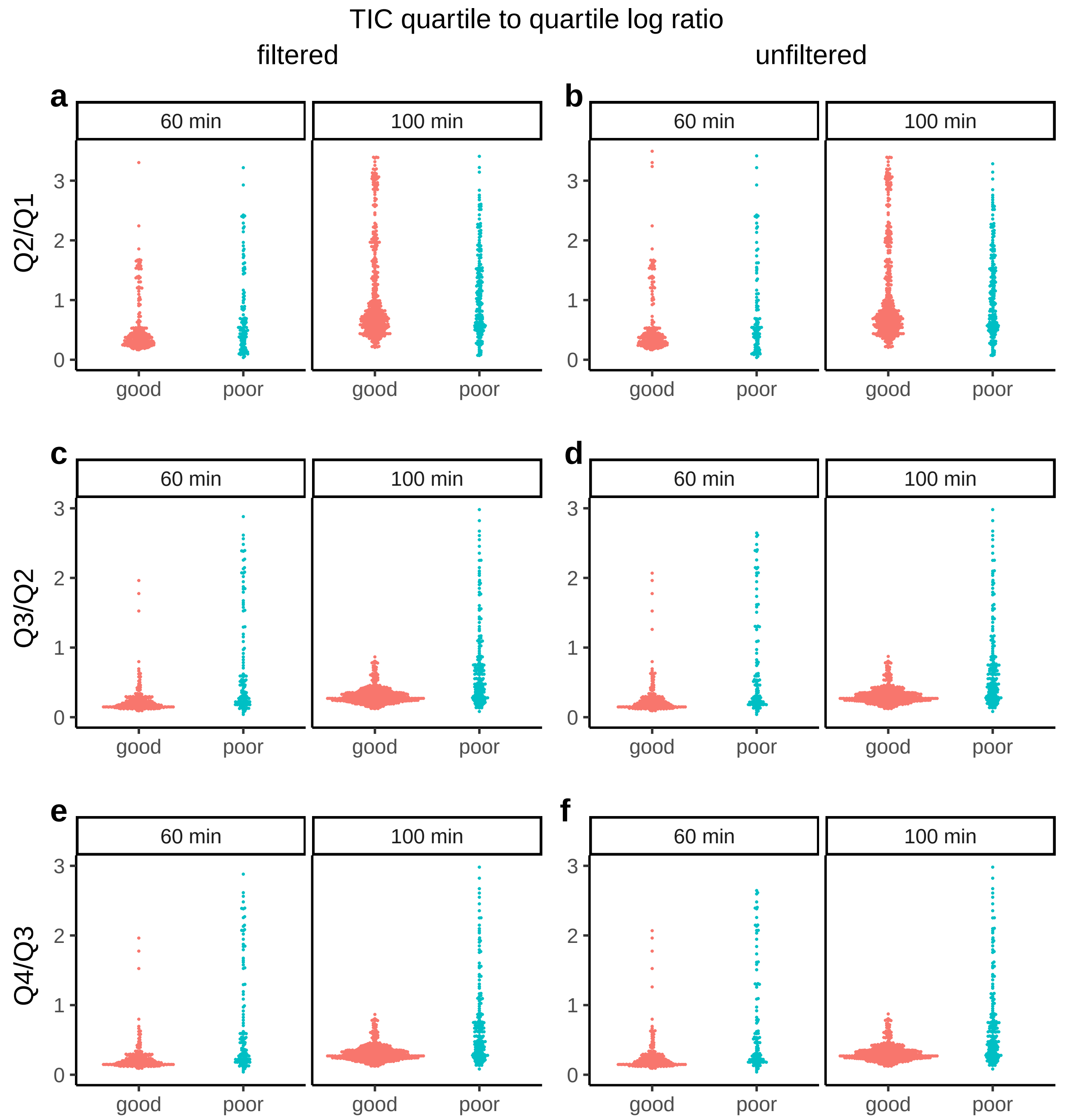

Figure S7: Quality metrics by `MsQuality`: TIC quartile to quartile log ratio (`ticQuartileToQuartileLogRatio`). The `MsQuality` metrics are calculated from filtered and unfiltered MS1 spectra. One data point is obtained per MS1 measurement run and the data points are displayed as beeswarm plots stratified for high-quality and low-quality measurements as classified in Amidan et al. [2014]. (a) log ratio of quartile 2 to quartile 1 for filtered MS1 spectra. (b) log ratio of quartile 2 to quartile 1 for unfiltered MS1 spectra. (c) log ratio of quartile 3 to quartile 2 for filtered MS1 spectra. (d) log ratio of quartile 3 to quartile 2 for unfiltered MS1 spectra. (e) log ratio of quartile 4 to quartile 3 for filtered MS1 spectra. (f) log ratio of quartile 4 to quartile 3 for unfiltered MS1 spectra.

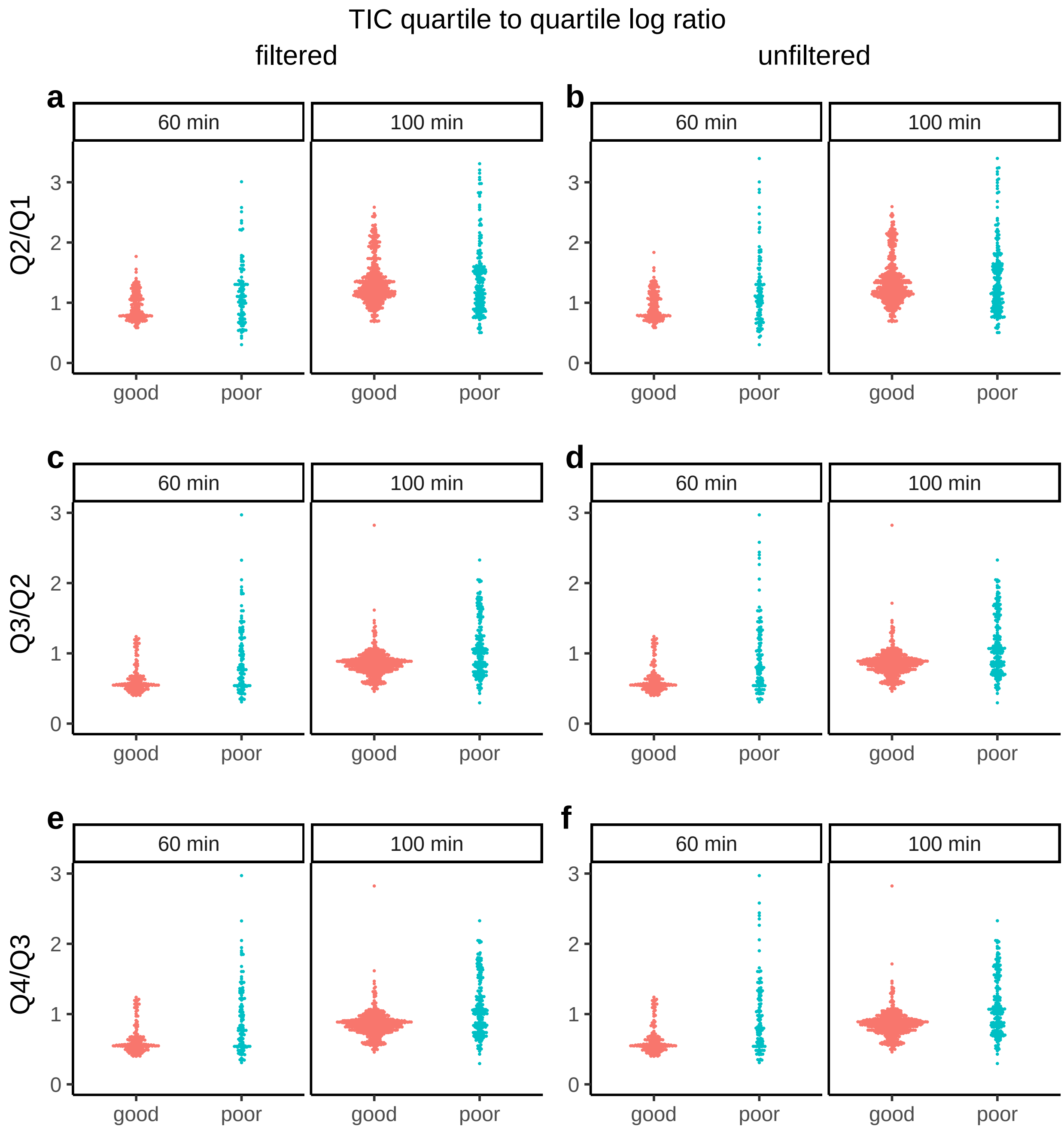

Figure S8: Quality metrics by `MsQuality`: TIC quartile to quartile log ratio (`ticQuartileToQuartileLogRatio`). The `MsQuality` metrics are calculated from filtered and unfiltered MS2 spectra. One data point is obtained per MS2 measurement run and the data points are displayed as beeswarm plots stratified for high-quality and low-quality measurements as classified in Amidan et al. [2014]. (a) log ratio of quartile 2 to quartile 1 for filtered MS2 spectra. (b) log ratio of quartile 2 to quartile 1 for unfiltered MS2 spectra. (c) log ratio of quartile 3 to quartile 2 for filtered MS2 spectra. (d) log ratio of quartile 3 to quartile 2 for unfiltered MS2 spectra. (e) log ratio of quartile 4 to quartile 3 for filtered MS2 spectra. (f) log ratio of quartile 4 to quartile 3 for unfiltered MS2 spectra.

The Figures S4, S5, and S6 demonstrate that the low-quality measurements (`poor`) have lower total ion current (TIC) values compared to high-quality measurements (`good`). The Figures serve as a visual check to differences in TIC values and aids in understanding the data quality of the measurements. It has to be pointed out that a further stratification (e.g. along the instrument type) might be helpful to further point out differences between the levels of data quality of the Amidan et al. [2014] data set.

The Figures S3, S7, and S8 on the other hand do not indicate differences between data quality and might not be indicative for the quality issues of the Amidan et al. [2014] data set.

5.3.1 Comparison to QuaMeter metrics

In the following, we will compare the `QuaMeter` metrics to the `MsQuality` metrics to check if `MsQuality` shows concordant results compared to `QuaMeter`. The `QuaMeter` metrics were calculated via the command line tool `bumbershoot` with `-MetricsType` set to `idfree`. The metric `IS-1A` was taken from the Supplemental Files of Amidan et al. [2014] since its calculation is not supported by the current version of `bumbershoot` (Chambers et al. [2012], Linux, 64-bit, gcc7-release-3_0_23220).

`QuaMeter` removes the entries of .mzML files with `defaultArrayLength=0` at any MS level. Thus, the metrics that were calculated by the .mzML files where the zero-length and zero-intensity entries were removed showed higher correlation compared to the unfiltered files. We provide flexibility to remove zero-length and zero-intensity entries by setting the argument `filterEmptySpectra` to `TRUE` or `FALSE` depending on the intended behavior.

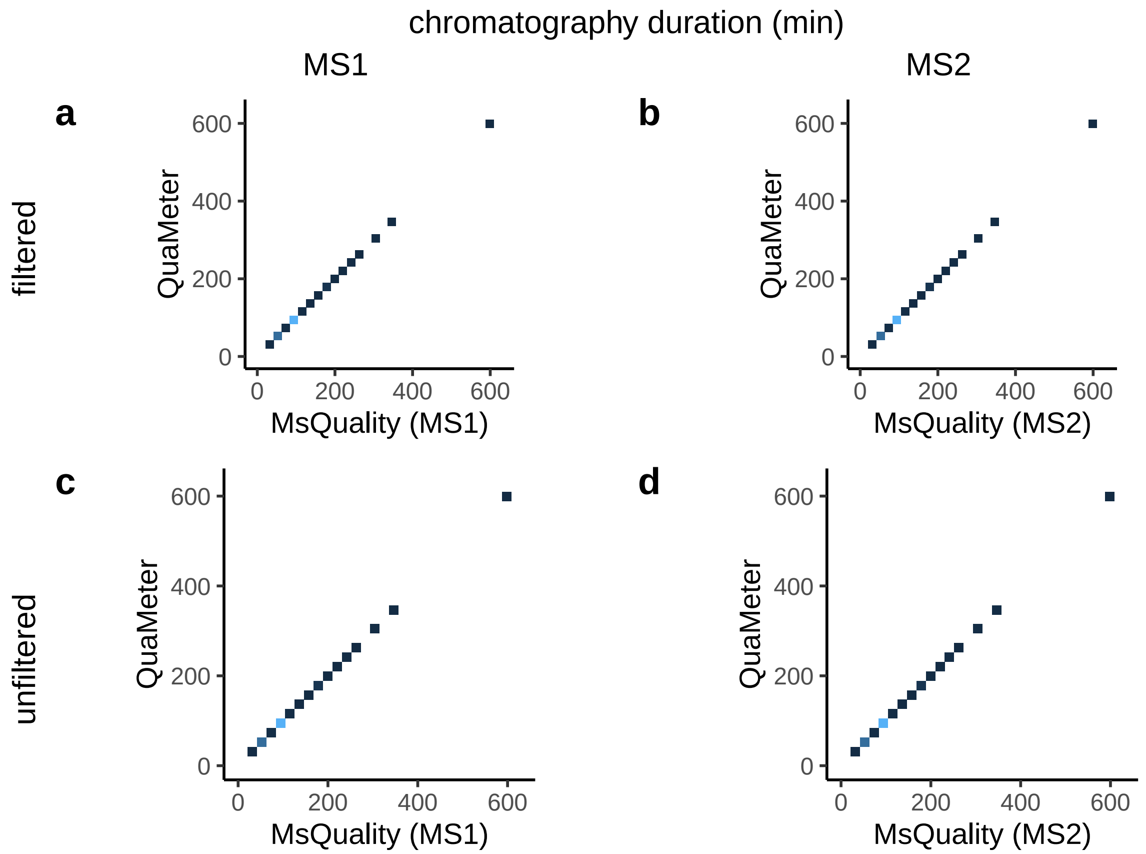

Figure S9: Comparison of quality metrics calculated by `MsQuality` and `QuaMeter`: chromatography duration (`chromatographyDuration`). The corresponding metric for `QuaMeter` is `RT-Duration` (no specification if the metric was calculated on MS1 and/or MS2 spectra). The `MsQuality` metrics are calculated from filtered and unfiltered MS1 and MS2 spectra. One data point is obtained per MS1 and MS2 measurement run and the data points are displayed as 2D densities. Brighter areas correspond to high 2D density areas. (a) chromatography duration for filtered MS1 spectra (`QuaMeter` metric: `RT-Duration`). (b) chromatography duration for unfiltered MS1 spectra (`QuaMeter` metric: `RT-Duration`). (c) chromatography duration for filtered MS2 spectra (`QuaMeter` metric: `RT-Duration`). (d) chromatography duration for unfiltered MS2 spectra (`QuaMeter` metric: `RT-Duration`).

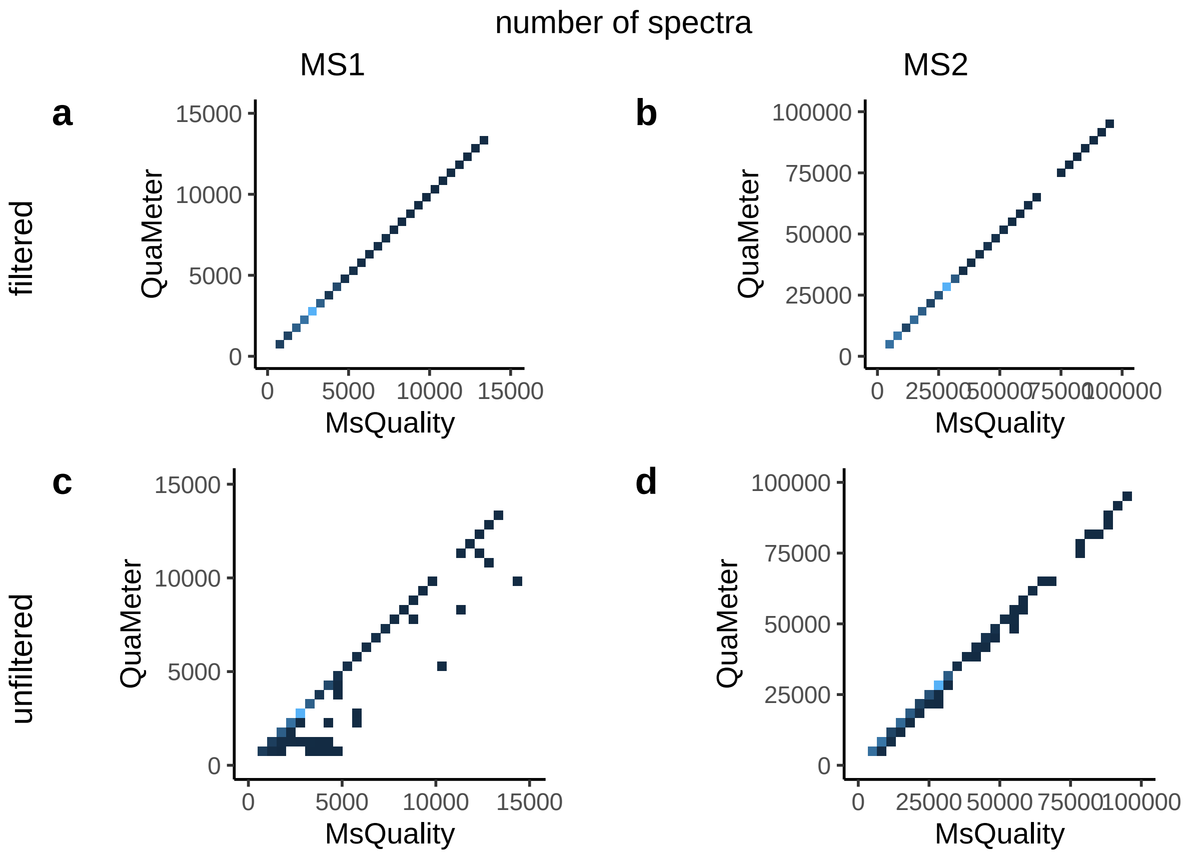

Figure S10: Comparison of quality metrics calculated by `MsQuality` and `QuaMeter`: Number of spectra (`numberSpectra`). The corresponding metrics for `QuaMeter` are `MS1-Count` and `MS2-Count`. The `MsQuality` metrics are calculated from filtered and unfiltered MS1 and MS2 spectra. One data point is obtained per MS1 and MS2 measurement run and the data points are displayed as 2D densities. Brighter areas correspond to high 2D density areas. (a) Number of filtered MS1 spectra (`QuaMeter` metric: `MS1-Count`). (b) Number of unfiltered MS1 spectra (`QuaMeter` metric: `MS1-Count`). (c) Number of filtered MS2 spectra (`QuaMeter` metric: `MS2-Count`). (b) Number of unfiltered MS2 spectra (`QuaMeter` metric: `MS2-Count`).

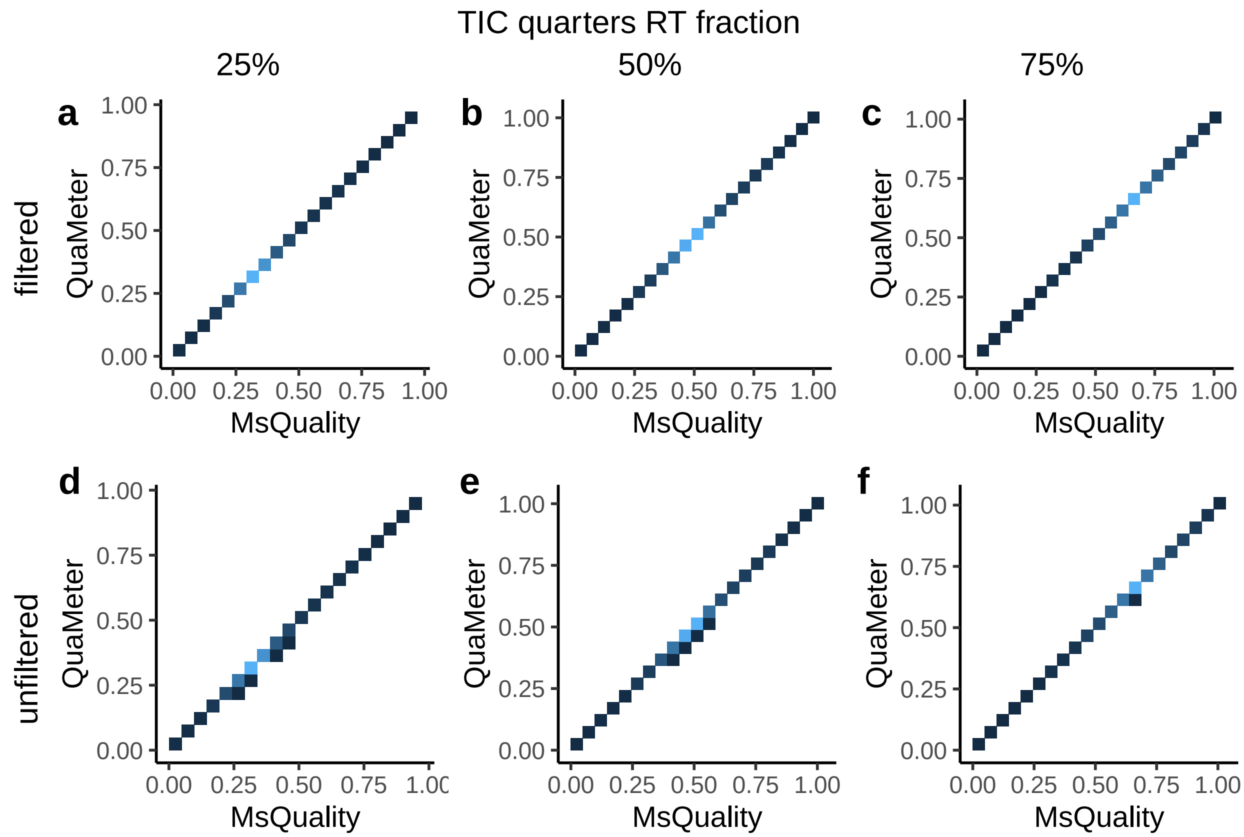

Figure S11: Comparison of quality metrics calculated by `MsQuality` and `QuaMeter`: TIC quarters RT fraction (`ticQuartersRtFraction`). The corresponding metrics for `QuaMeter` are `RT-TIC-Q1`, `RT-TIC-Q2`, and `RT-TIC-Q3` (no specification if these metrices were calculated on MS1 and/or MS2 spectra). The `MsQuality` metrics are calculated from filtered and unfiltered MS1 spectra. One data point is obtained per MS1 measurement run and the data points are displayed as 2D densities. Brighter areas correspond to high 2D density areas. (a) 25% quantile for filtered MS1 spectra (`QuaMeter` metric: `RT-TIC-Q1`). (b) 50% quantile for filtered MS1 spectra (`QuaMeter` metric: `RT-TIC-Q2`). (c) 75% quantile for filtered MS1 spectra (`QuaMeter` metric: `RT-TIC-Q3`). (d) 25% quantile for unfiltered MS1 spectra (`QuaMeter` metric: `RT-TIC-Q1`). (e) 50% quantile for unfiltered MS1 spectra (`QuaMeter` metric: `RT-TIC-Q2`). (f) 75% quantile for unfiltered MS1 spectra (`QuaMeter` metric: `RT-TIC-Q3`).

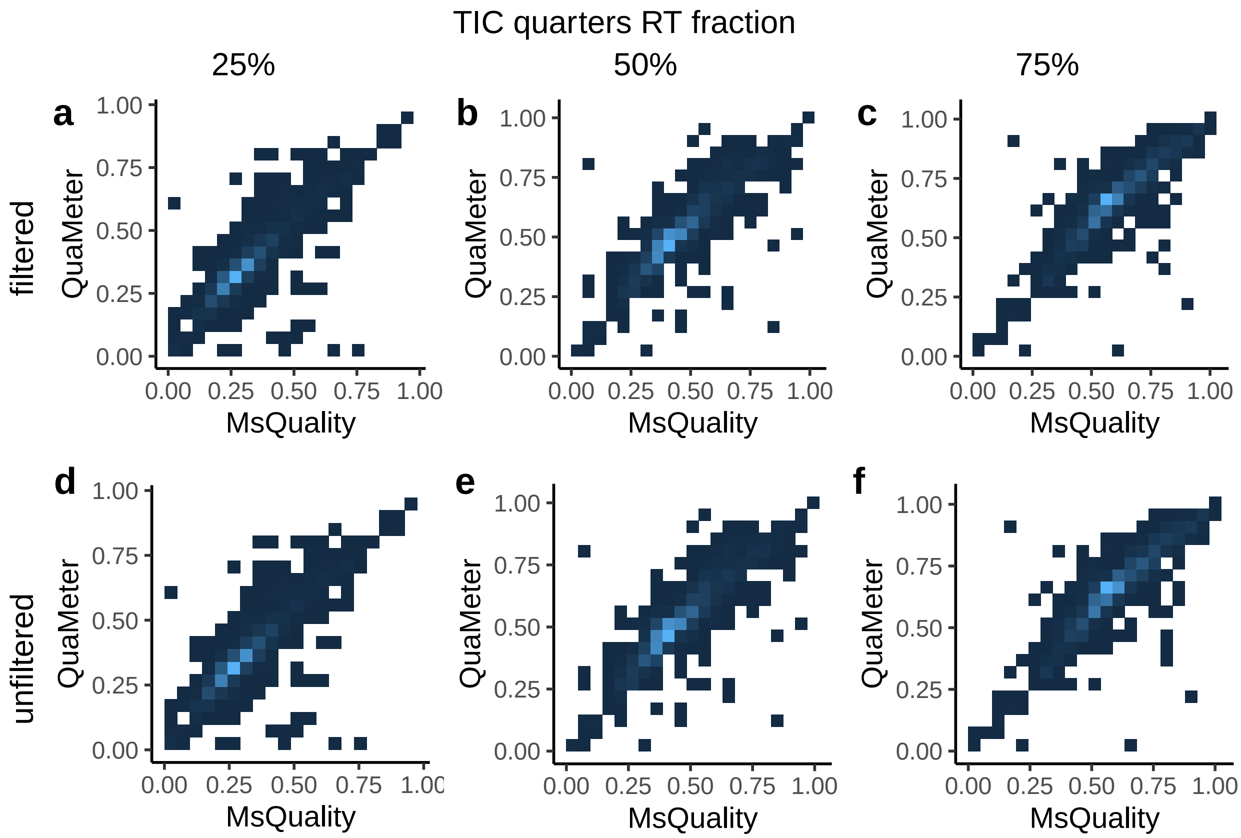

Figure S12: Comparison of quality metrics calculated by `MsQuality` and `QuaMeter`: TIC quarters RT fraction (`ticQuartersRtFraction`). The corresponding metrics for `QuaMeter` are `RT-TIC-Q1`, `RT-TIC-Q2`, and `RT-TIC-Q3` (no specification if these metrices were calculated on MS1 and/or MS2 spectra). The `MsQuality` metrics are calculated from filtered and unfiltered MS2 spectra. One data point is obtained per MS2 measurement run and the data points are displayed as 2D densities. Brighter areas correspond to high 2D density areas. (a) 25% quantile for filtered MS2 spectra (`QuaMeter` metric: `RT-TIC-Q1`). (b) 50% quantile for filtered MS2 spectra (`QuaMeter` metric: `RT-TIC-Q2`). (c) 75% quantile for filtered MS2 spectra (`QuaMeter` metric: `RT-TIC-Q3`). (d) 25% quantile for unfiltered MS2 spectra (`QuaMeter` metric: `RT-TIC-Q1`). (e) 50% quantile for unfiltered MS2 spectra (`QuaMeter` metric: `RT-TIC-Q2`). (f) 75% quantile for unfiltered MS2 spectra (`QuaMeter` metric: `RT-TIC-Q3`).

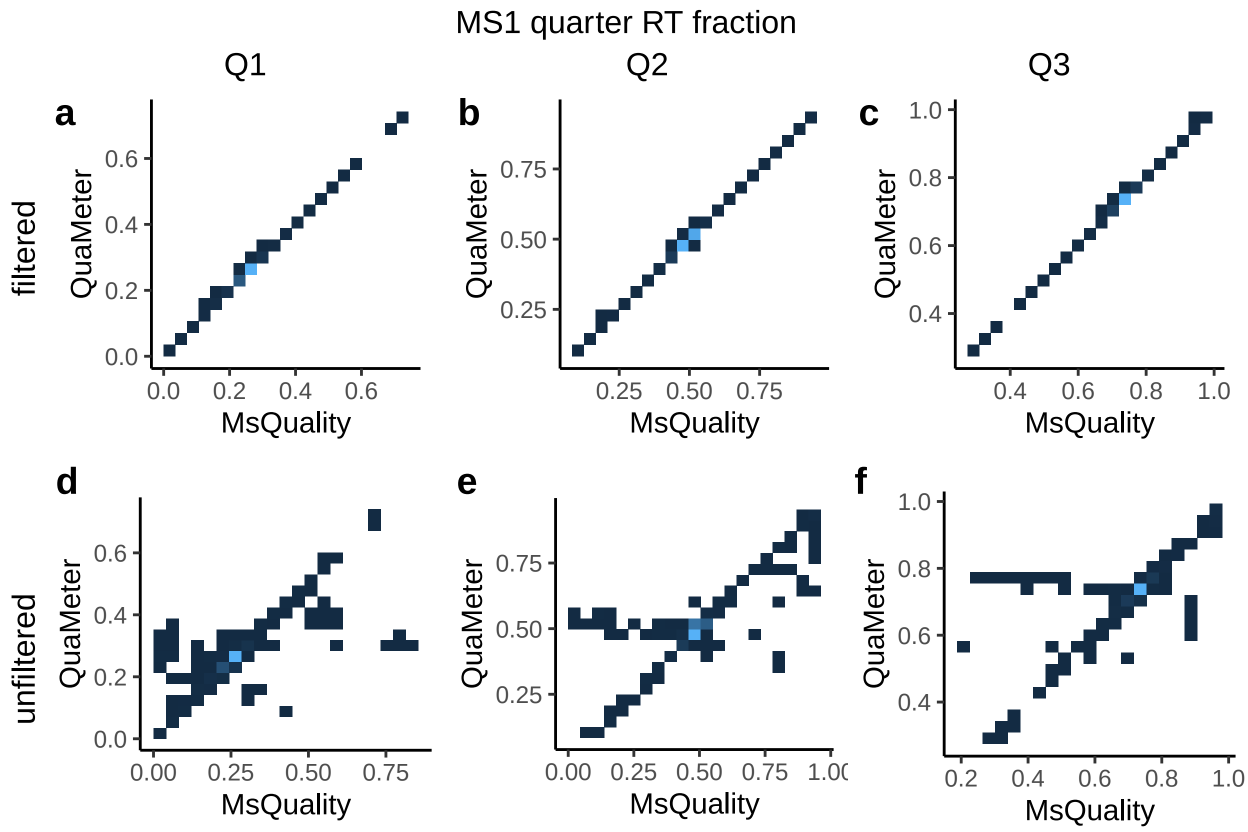

Figure S13: Comparison of quality metrics calculated by `MsQuality` and `QuaMeter`: MS1 quarter RT fraction (`rtOverMsQuarters`). The corresponding metrics for `QuaMeter` are `RT-MS-Q1`, `RT-MS-Q2`, and `RT-MS-Q3`. The `MsQuality` metrics are calculated from filtered and unfiltered MS1 spectra. One data point is obtained per MS1 measurement run and the data points are displayed as 2D densities. Brighter areas correspond to high 2D density areas. (a) Quarter 1 for filtered MS1 spectra (`QuaMeter` metric: `RT-MS-Q1`). (b) Quarter 2 for filtered MS1 spectra (`QuaMeter` metric: `RT-MS-Q2`). (c) Quarter 3 for filtered MS1 spectra (`QuaMeter` metric: `RT-MS-Q3`). (d) Quarter 1 for unfiltered MS1 spectra (`QuaMeter` metric: `RT-MS-Q1`). (e) Quarter 2 for unfiltered MS1 spectra (`QuaMeter` metric: `RT-MS-Q2`). (f) Quarter 3 for unfiltered MS1 spectra (`QuaMeter` metric: `RT-MS-Q2`). Q: quarter.

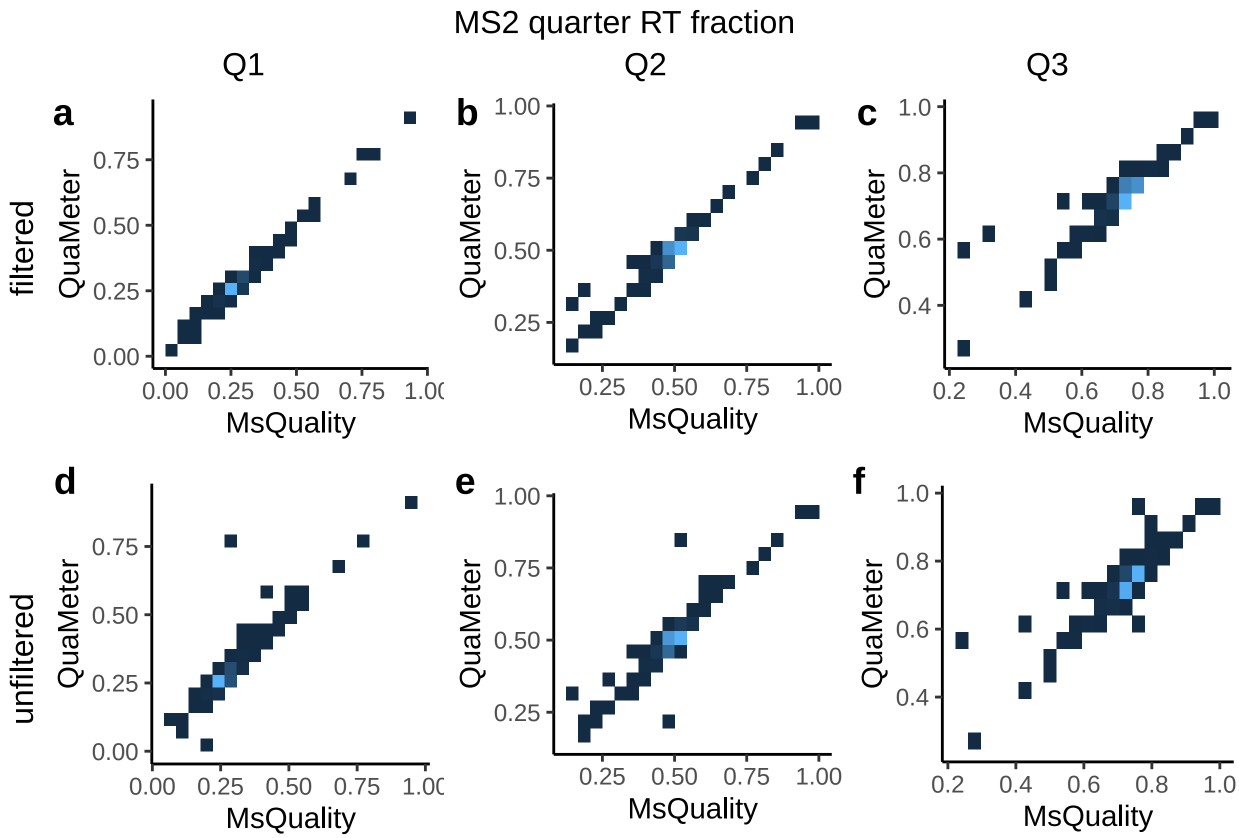

Figure S14: Comparison of quality metrics calculated by `MsQuality` and `QuaMeter`: MS2 quarter RT fraction (`rtOverMsQuarters`). The corresponding metrics for `QuaMeter` are `RT-MSMS-Q1`, `RT-MSMS-Q2`, and `RT-MSMS-Q3`. The `MsQuality` metrics are calculated from filtered and unfiltered MS2 spectra. One data point is obtained per MS2 measurement run and the data points are displayed as 2D densities. Brighter areas correspond to high 2D density areas. (a) Quarter 1 for filtered MS2 spectra (`QuaMeter` metric: `RT-MSMS-Q1`). (b) Quarter 2 for filtered MS2 spectra (`QuaMeter` metric: `RT-MSMS-Q2`). (c) Quarter 3 for filtered MS2 spectra (`QuaMeter` metric: `RT-MSMS-Q3`). (d) Quarter 1 for unfiltered MS2 spectra (`QuaMeter` metric: `RT-MSMS-Q1`). (e) Quarter 2 for unfiltered MS2 spectra (`QuaMeter` metric: `RT-MSMS-Q2`). (f) Quarter 3 for unfiltered MS2 spectra (`QuaMeter` metric: `RT-MSMS-Q3`). Q: quarter.

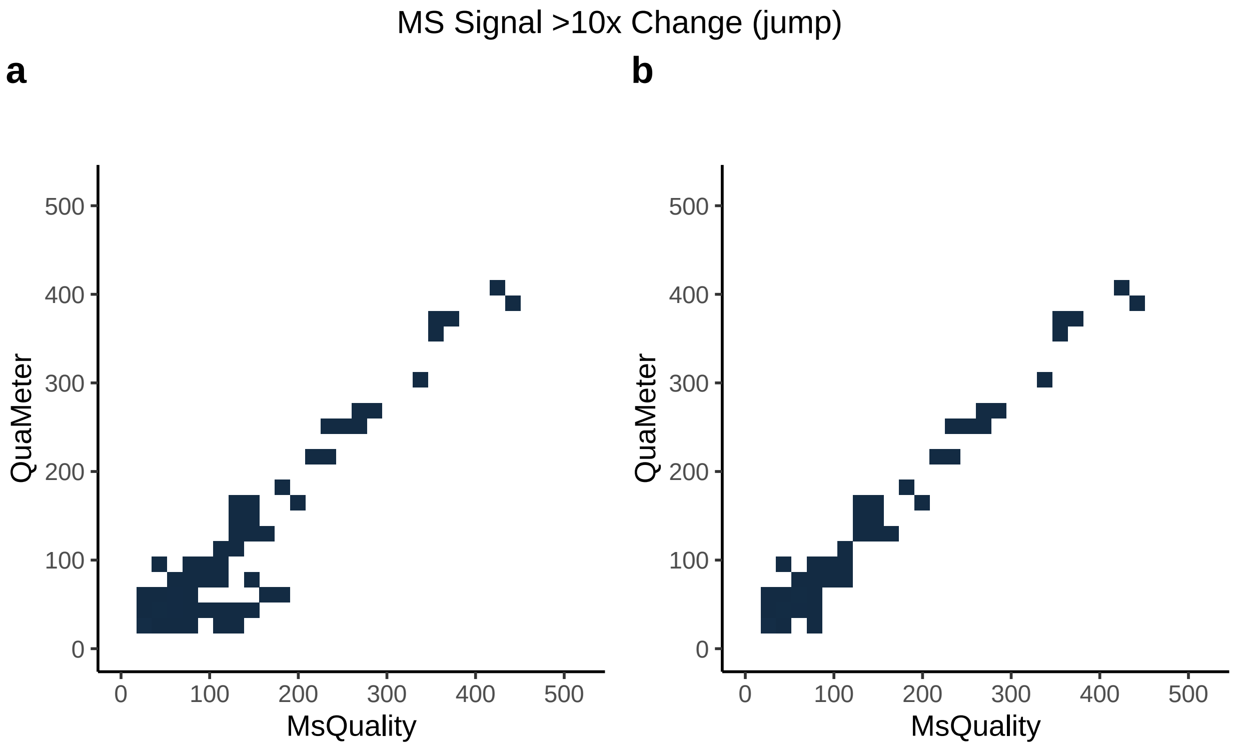

Figure S15: Comparison of quality metrics calculated by `MsQuality` and `QuaMeter`: MS Signal >10x Change (jump, `msSignal10xChange`). The corresponding metric for `QuaMeter` is `IS-1A`. The metrics are calculated from filtered and unfiltered MS1 spectra. One data point is obtained per MS1 measurement run and the data points are displayed as 2D densities. Brighter areas correspond to high 2D density areas. (a) Jumps for filtered MS1 spectra (`QuaMeter` metric: `IS_1A`). (b) Jumps for unfiltered MS1 spectra (`QuaMeter` metric: `IS-1A`).

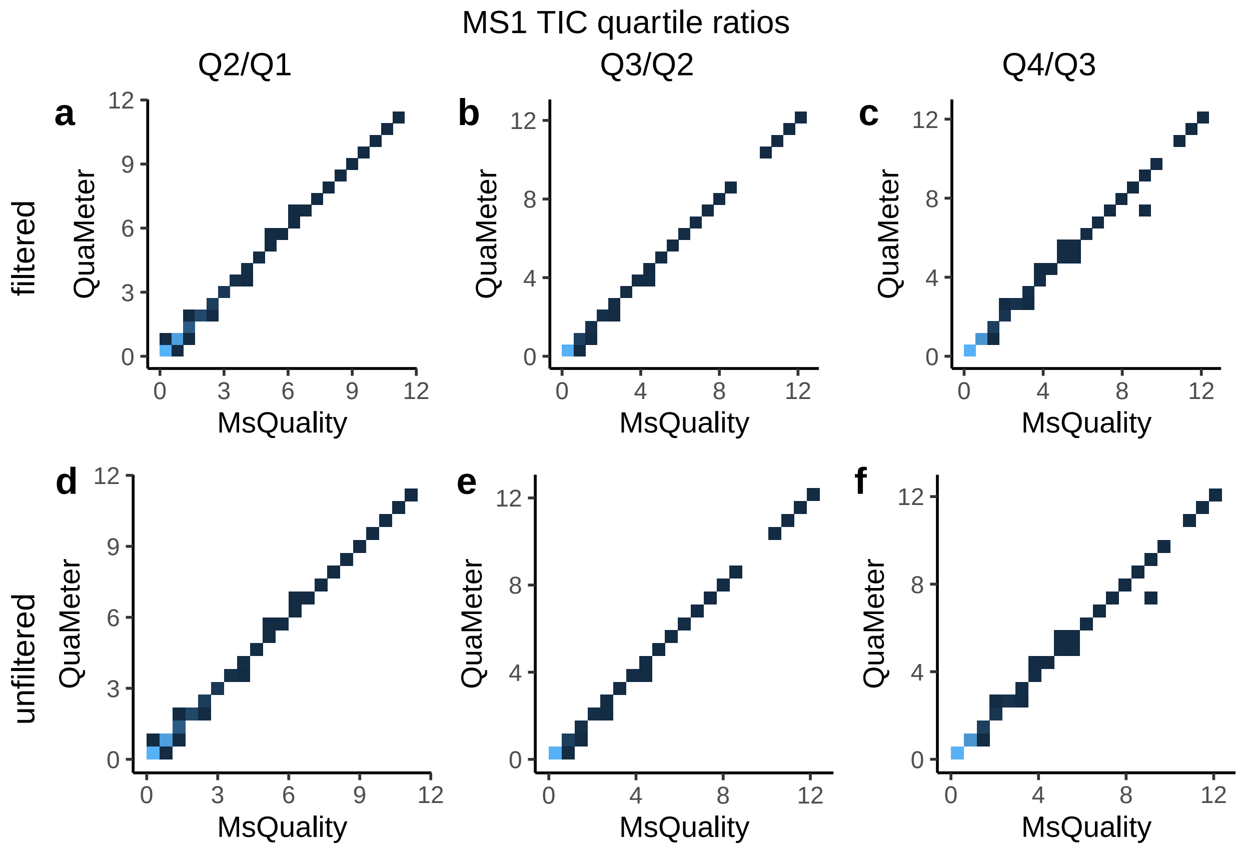

Figure S16: Comparison of quality metrics calculated by `MsQuality` and `QuaMeter`: MS1 TIC quartile ratios (`ticQuartileToQuartileLogRatio`). The corresponding metrics for `QuaMeter` are `MS1-TIC-Q2`, `MS1-TIC-Q3`, and `MS1-TIC-Q4`. The `MsQuality` metrics are calculated from filtered and unfiltered MS1 spectra. One data point is obtained per MS1 measurement run and the data points are displayed as 2D densities. Brighter areas correspond to high 2D density areas. (a) Q2/Q1 log ratio for filtered MS1 spectra (`QuaMeter` metric: `MS1-TIC-Q2`). (b) Q3/Q2 log ratio for filtered MS1 spectra (`QuaMeter` metric: `MS1-TIC-Q3`). (c) Q4/Q3 log ratio for filtered MS1 spectra (`QuaMeter` metric: `MS1-TIC-Q4`). (d) Q2/Q1 log ratio for unfiltered MS1 spectra (`QuaMeter` metric: `MS1-TIC-Q2`). (e) Q3/Q2 log ratio for unfiltered MS1 spectra (`QuaMeter` metric: `MS1-TIC-Q3`). (f) Q4/Q3 log ratio for unfiltered MS1 spectra (`QuaMeter` metric: `MS1-TIC-Q4`).

The Figures S9, S10, S11, S12, S13, S14, S15, and S16 indicate that the `QuaMeter` and `MsQuality` generally compute similar values. This is shown by values that show high correlation (points locate close to the identity line within the scatter plots). We provide in Table S1 the Pearson and Spearman correlation coefficients of the `MsQuality` metrics with their corresponding `QuaMeter` metrics. Table S2 is a higher-level analysis regarding the quantiles of Pearson and Spearman correlation coefficients between the `MsQuality` and `QuaMeter` metrics. This analysis supports the observation that `MsQuality` calculates highly similar metric values as 75% of the metrics show Pearson/Spearman coefficients of 0.81/0.87 or higher.

Table S1: Pearson and Spearman correlation coefficients between `MsQuality` and precalculated `QuaMeter` metrics.

MsQuality	QuaMeter	MS level	Pearson coef. (filtered)	Spearman coef. (filtered)	Pearson coef.	Spearman coef.
chromatographyDuration	RT-Duration	1	1	1	1	0.998
chromatographyDuration	RT-Duration	2	1	1	1	0.998
numberSpectra	MS1-Count	1	1	1	0.968	0.972
numberSpectra	MS2-Count	2	1	1	1	0.999
ticQuartersRtFraction.25%	RT-TIC-Q1	1	1	1	1	1
ticQuartersRtFraction.25%	RT-TIC-Q1	2	0.874	0.89	0.874	0.89
ticQuartersRtFraction.50%	RT-TIC-Q2	1	1	1	1	1
ticQuartersRtFraction.50%	RT-TIC-Q2	2	0.901	0.899	0.901	0.899
ticQuartersRtFraction.75%	RT-TIC-Q3	1	1	1	1	1
ticQuartersRtFraction.75%	RT-TIC-Q3	2	0.912	0.906	0.912	0.906
rtOverMsQuarters.Quarter1	RT-MS-Q1	1	1	1	0.734	0.908
rtOverMsQuarters.Quarter1	RT-MSMS-Q1	2	0.997	0.999	0.953	0.985
rtOverMsQuarters.Quarter2	RT-MS-Q2	1	1	1	0.806	0.9
rtOverMsQuarters.Quarter2	RT-MSMS-Q2	2	0.989	0.995	0.964	0.978
rtOverMsQuarters.Quarter3	RT-MS-Q3	1	1	1	0.812	0.916
rtOverMsQuarters.Quarter3	RT-MSMS-Q3	2	0.966	0.986	0.949	0.974
msSignal10xChange	IS-1A	1	0.666	0.858	0.687	0.838
ticQuartileToQuartileLogRatio.Q2/Q1	MS1-TIC-Q2	1	1	1	0.949	0.981
ticQuartileToQuartileLogRatio.Q3/Q2	MS1-TIC-Q3	1	1	1	0.934	0.984
ticQuartileToQuartileLogRatio.Q4/Q3	MS1-TIC-Q4	1	1	1	0.953	0.991

Table S2: Quantiles for Pearson and Spearman correlation coefficients for `MsQuality` and `QuaMeter` metrics. For the filtered spectra in `MsQuality`, 75% of the `MsQuality` metrics showed Pearson/Spearman correlation coefficients over 0.84/0.88, 50% over 0.95/0.96, and 25% over 1.0/1.0 to the `QuaMeter` metrics. For the unfiltered spectra in `MsQuality`, 75% of the `MsQuality` metrics showed Pearson/Spearman correlation coefficients over 0.79/0.86, 50% over 0.89/0.90, and 25% over 0.96/0.97 to the `QuaMeter` metrics.

Quantile	Pearson coef. (filtered)	Spearman coef. (filtered)	Pearson coef.	Spearman coef.
0%	0.67	0.86	0.69	0.84
10%	0.90	0.90	0.80	0.90
20%	0.96	0.97	0.86	0.90
25%	0.98	0.99	0.89	0.91
30%	0.99	1.00	0.91	0.91
40%	1.00	1.00	0.94	0.97
50%	1.00	1.00	0.95	0.98
60%	1.00	1.00	0.96	0.98
70%	1.00	1.00	0.98	0.99
75%	1.00	1.00	1.00	1.00
80%	1.00	1.00	1.00	1.00
90%	1.00	1.00	1.00	1.00
100%	1.00	1.00	1.00	1.00

5.4 Performance under parallelization

Similar to the above-mentioned analysis using the flow injection analysis, an important aspect, especially when dealing with large amount of data, is scalability and performance when computing the quality metric.

We measure the time it takes to calculate the quality metrics under parallelization of the tasks on 1, 2, 4, 8, and 16 workers using the `microbenchmark` package. For computational reasons we limit the calculation to the first 500 .mzML files. The operations were executed within a (high-performance) computing environment (31 cores, 128 GB RAM pool for all cores).

```r
path <- "/scratch/naake/Amidan2014"
fls <- dir(path, full.names = TRUE, recursive = TRUE, pattern = "mzML") |>
    unique()
fls <- fls[1:500]
sps_mb <- sps[sps$dataOrigin %in% fls]

metrics <- c("chromatographyDuration", "ticQuartersRtFraction",
    "rtOverMsQuarters", "ticQuartileToQuartileLogRatio", "numberSpectra",
    "numberEmptyScans", "medianPrecursorMz", "rtIqr", "rtIqrRate",
    "areaUnderTic", "areaUnderTicRtQuantiles", "medianTicRtIqr",
    "medianTicOfRtRange", "mzAcquisitionRange", "rtAcquisitionRange",
    "precursorIntensityRange", "precursorIntensityQuartiles",
    "precursorIntensityMean", "precursorIntensitySd",
    "msSignal10xChange", "ratioCharge1over2", "ratioCharge3over2",
    "ratioCharge4over2", "meanCharge", "medianCharge")

df_mb <- microbenchmark(
    workers_1 = calculateMetricsFromSpectra(spectra = sps_mb,
        metrics = metrics, BPPARAM = MulticoreParam(workers = 1)),
    workers_2 = calculateMetricsFromSpectra(spectra = sps_mb,
        metrics = metrics, BPPARAM = MulticoreParam(workers = 2)),
    workers_4 = calculateMetricsFromSpectra(spectra = sps_mb,
        metrics = metrics, BPPARAM = MulticoreParam(workers = 4)),
    workers_8 = calculateMetricsFromSpectra(spectra = sps_mb,
        metrics = metrics, BPPARAM = MulticoreParam(workers = 8)),
    workers_16 = calculateMetricsFromSpectra(spectra = sps_mb,
        metrics = metrics, BPPARAM = MulticoreParam(workers = 16)),
    times = 32L, control = list(warmup = 2), check = "equal"
)
```

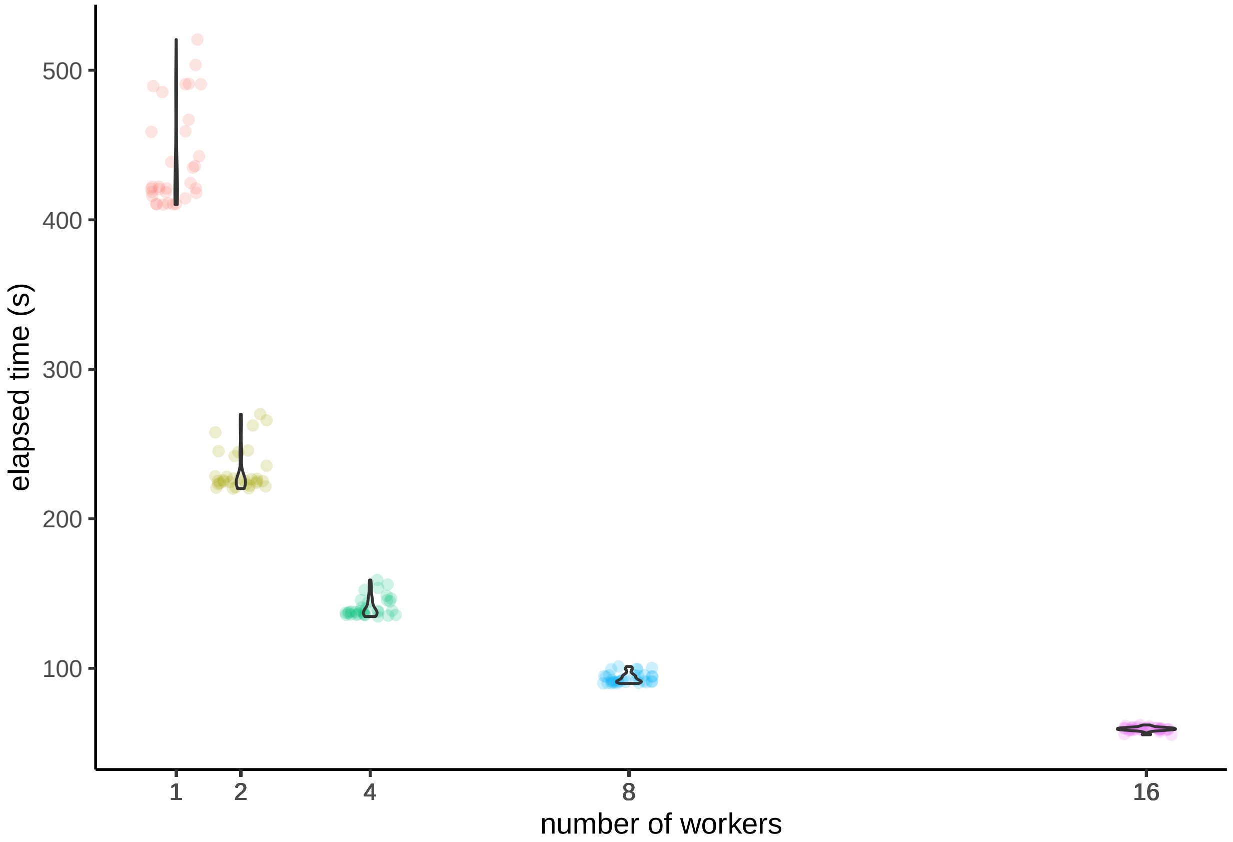

Figure S17: Execution time for the calculation of quality metrics of the data set of Amidan et al. [2014] under parallelization (1, 2, 4, 8, and 16 workers).

The `microbenchmark` package was used to accurately measure the performance improvements achieved by parallelization (Figure S17). By parallelizing the calculation of the quality metrics across multiple workers, it is possible to significantly reduce the execution time.

6 Session info

Information on the attached packages.

```
## R version 4.2.2 (2022-10-31 ucrt)
## Platform: x86_64-w64-mingw32/x64 (64-bit)
## Running under: Windows 10 x64 (build 19044)
##
## Matrix products: default
##
## locale:
## [1] LC_COLLATE=English_Germany.utf8  LC_CTYPE=English_Germany.utf8
## [3] LC_MONETARY=English_Germany.utf8 LC_NUMERIC=C
## [5] LC_TIME=English_Germany.utf8
##
## attached base packages:
## [1] stats4    stats     graphics  grDevices utils     datasets  methods
## [8] base
##
## other attached packages:
##  [1] patchwork_1.1.3       BiocFileCache_2.6.1   dbplyr_2.3.3
##  [4] curl_5.0.1            tidyr_1.3.0           tibble_3.2.1
##  [7] stringr_1.5.0         readxl_1.4.3          dplyr_1.1.2
## [10] ggpubr_0.6.0          ggbeeswarm_0.7.2      ggplot2_3.4.3
## [13] microbenchmark_1.4.10 MsQuality_1.1.3       mzR_2.32.0
## [16] Rcpp_1.0.10           Spectra_1.8.3         ProtGenerics_1.30.0
## [19] BiocParallel_1.32.6   S4Vectors_0.36.2      BiocGenerics_0.44.0
## [22] knitr_1.42            BiocStyle_2.26.0
```

References

B.G. Amidan, D.J. Orton, B.L. Lamarche, M.E. Monroe, R.J. Moore, A.M. Venzin, R.D. Smith, L.H. Sego, M.F. Tardiff, and S.H. Payne. Signatures for mass spectrometry data quality. *Proteome Research*, 13:2215–2222, 2014. doi: 10.1021/pr401143e.

M.S. Bereman. Tools for monitoring system suitability in lc ms/ms centric proteomic experiments. *Proteomics*, 15:891–902, 2015. doi: 10.1002/pmic.201400373.

W. Bittremieux, D. Valkenborg, L. Martens, and K. Laukens. Computational quality control tools for mass spectrometry proteomics. *Proteomics*, 17:1–11, 2017. doi: 10.1002/pmic.201600159.

M.C. Chambers, B. Maclean, R. Burke, D. Amodei, D.L. Ruderman, S. Neumann, L. Gatto, B. Fischer, B. Pratt, J. Egertson, K. Hoff, D. Kessner, T. Tasman, N. Shulman, B. Frewen, T.A. Baker, M.-Y. Brusniak, C. Paulse, D. Creasy, L. Flashner, K. Kani, C. Moulding, S.L. Seymour, L.M. Nuwaysir, B. Lefebvre, F. Kuhlmann, J. Roark, P. Rainer, S. Detlev, T. Hemenway, A. Huhmer, J. Langridge, B. Connolly, T. Chadick, K. Holly, J. Eckels, E.W. Deutsch, R.L. Moritz, J.E. Katz, D.B. Agus, M. MacCoss, D.L. Tabb, and P. Mallick. A cross-platform toolkit for mass spectrometry and proteomics. *Nature Biotechnology*, 30: 918–920, 2012. doi: 10.1038/nbt.2377.

S. Cherkaoui, S. Durot, J. Bradley, S. Critchlow, S. Dubuis, M.M. Masiero, R. Wegmann, B. Snijder, A. Othman, C. Bendtsen, and N. Zamboni. A functional analysis of 180 cancer cell lines reveals conserved intrinsic metabolic programs. *Molecular Systems Biology*, 18: e11033, 2022. doi: 10.15252/msb.202211033.

T. Köcher, P. Pichler, R. Swart, and K. Mechtler. Quality control in lc-ms/ms. *Proteomics and Systems Biology*, 11:1026–1030, 2011. doi: 10.1002/pmic.201000578.

T. Naake and W. Huber. Matrixqcvis: shiny-based interactive data quality exploration for omics data. *Bioinformatics*, 38:1181–1182, 2022. doi: 10.1093/bioinformatics/btab748.

A.G. Paulovich, D. Billheimer, A.-J. L. Ham, L. Vega-Montoto, P.A. Rudnick, D.L. Tabb, P. Wang, D.M. Blackman, D.M. Bunk, H.L. Cardasis, K.R. Clauser, C.R. Kinsinger, B. Schilling, T.J. Tegeler, A.M. Variyath, M. Wang, J.R. Whiteaker, L.J. Zimmerman, D. Fenyo, S.A. Carr, S.J. Fisher, B.W. Gibson, M. Mesri, T.A. Neubert, F.E. Regnier, H. Rodriguez, C. Spiegelman, S.E. Stein, P. Tempst, and D.C. Liebler. Interlaboratory study characterizing a yeast performance standard for benchmarking lc-ms platform performance. *Molecular & Cellular Proteomics*, 9:242–254, 2010. doi: 10.1074/mcp.M900222-MCP200.
